# Longitudinal analysis of acute and convalescent B cell responses in a human primary dengue serotype 2 infection model

**DOI:** 10.1016/j.ebiom.2019.02.060

**Published:** 2019-03-08

**Authors:** Usha K. Nivarthi, Huy A. Tu, Matthew J. Delacruz, Jesica Swanstrom, Bhumi Patel, Anna P. Durbin, Stephen S. Whitehead, Kristen K. Pierce, Beth D. Kirkpatrick, Ralph S. Baric, Ngan Nguyen, Daniel E. Emerling, Aravinda M. de Silva, Sean A. Diehl

**Affiliations:** a Department of Microbiology and Immunology, University of North Carolina School of Medicine, Chapel Hill, North Carolina 27599, USA; b Department of Microbiology and Molecular Genetics, Vaccine Testing Center, Larner College of Medicine, University of Vermont, Burlington, VT 05405, USA; c Cellular, Molecular, and Biomedical Sciences Graduate Program, University of Vermont, Burlington, VT 05405, USA; d Department of International Health, Center for Immunization Research, Johns Hopkins Bloomberg School of Public Health, Baltimore, MD 21205, USA; e Laboratory of Infectious Diseases, NIAID, National Institutes of Health, Bethesda, MD 20892, USA; f Atreca, Inc. Redwood City, California 94063, USA

**Keywords:** Dengue, Viral infection, Humoral immunity, Antibody

## Abstract

**Background:**

Acute viral infections induce a rapid and transient increase in antibody-secreting plasmablasts. At convalescence, memory B cells (MBC) and long-lived plasma cells (LLPC) are responsible for long-term humoral immunity. Following an acute viral infection, the specific properties and relationships between antibodies produced by these B cell compartments are poorly understood.

**Methods:**

We utilized a controlled human challenge model of primary dengue virus serotype 2 (DENV2) infection to study acute and convalescent B-cell responses.

**Findings:**

The level of DENV2 replication was correlated with the magnitude of the plasmablast response. Functional analysis of plasmablast-derived monoclonal antibodies showed that the DENV2-specific response was dominated by cells producing DENV2 serotype-specific antibodies. DENV2-neutralizing antibodies targeted quaternary structure epitopes centered on domain III of the viral envelope protein (EDIII). Functional analysis of MBC and serum antibodies from the same subjects six months post-challenge revealed maintenance of the serotype-specific response in both compartments. The serum response mainly targeted DENV2 serotype-specific epitopes on EDIII.

**Interpretation:**

Our data suggest overall functional alignment of DENV2-specific responses from the plasmablast, through the MBC and LLPC compartments following primary DENV2 inflection. These results provide enhanced resolution of the temporal and specificity of the B cell compartment in viral infection and serve as framework for evaluation of B cell responses in challenge models.

**Funding:**

This study was supported by the Bill and Melinda Gates Foundation and the National Institutes of Health.

## Highlights

• rDEN2Δ30 virus induced plasmablasts two weeks after infection of flavivirus-naïve human subjects.
• Expanded plasmablast lineages produced type-specific antibodies including neutralizers to quaternary E protein epitopes
• rDEN2Δ30-induced memory B cells retained functional, but not clonal type-specific overlap with the plasmablast response
• DENV2-specific antibodies present during convalescence after rDEN2Δ30 infection are highly type-specific.
• The rDEN2Δ30 challenge model is a framework against which to evaluate B cell responses to dengue natural infection and vaccination.

**Research in context**

**Evidence before this study**

Primary natural dengue infections are hypothesized to afford lifelong protection against the infecting serotype, in part, by production of antibodies specific to that serotype, but the ontogeny of this response at the level of B cells is not defined. Accurately determining infection status in and acquiring longitudinal samples from natural primary infection can be problematic. For our study we sought to utilize a controlled human infection model for dengue to define the specificity of the B cell response to primary dengue 2 infection from the acute stage through convalescence. At the onset of our study in 2015 we searched Pubmed (language filter off) for “dengue type 2 infection B cells” and retrieved 15 entries, none of which utilized a controlled human infection model for dengue.

**Value added**

Our work is the first to clearly show the origins and maintenance of serotype-specificity of the B cell response to primary dengue infection in healthy naïve subjects.

**Implications of all available evidence**

These findings are relevant to human health because the four serotypes of dengue are a major global health threat. A dengue vaccine that protects against all four serotypes in flavivirus-naïve individuals is urgently needed. These results serve as a baseline against which to evaluate dengue vaccine responses in flavivirus-naïve subjects both in terms of cellular response kinetics, magnitude, and specificity.

Alt-text: Unlabelled Box

## 1 Introduction

Dengue viruses (DENV) transmitted by mosquito vectors are the etiological agents of dengue fever and dengue hemorrhagic fever [1,2]. It is estimated that DENVs cause 390 million infections annually, mostly in the tropical and subtropical regions of the world [3]. With >100 million apparent cases, DENV infections are the leading cause of pediatric hospitalization in many afflicted countries.

The DENV complex consists of four closely related serotypes (DENV1–4). Following a primary natural DENV infection, high levels of circulating homotypic neutralizing antibodies against the infecting serotype are elicited [4] and maintained for decades [5], suggesting a contribution to lifelong protection against the infecting serotype. Serotype cross-reactive antibodies also elicited by primary infection are throught to provide transient cross-protection against secondary heterotypic infection with a new serotype [6]. More remote (years) secondary heterotypic infections are associated with a greater risk of developing severe dengue hemorrhagic fever [[7], [8], [9], [10], [11]]. The increased risk of severe disease in secondary cases appears to be driven, at least in part, by cross-reactive, non-neutralizing antibodies that can enhance viral infection [12]. The temporal relationship between early and late B cell responses and maintenance of serotype specificity following primary DENV infection is incompletely understood.

Plasmablasts are an antibody-secreting B cell population present at low levels in healthy peripheral blood but can increase dramatically in number following infection or vaccination [[13], [14], [15], [16], [17], [18]]. High levels of plasmablasts have been documented during acute ongoing DENV infection [[19], [20], [21]]. Later after recovery, long-term protective antibody responses are maintained for many years by antigen-specific memory B cells (MBC) and long-lived plasma cells (LLPC) [[22], [23], [24]]. Approximately 0.1–0.5% of the MBC in peripheral blood produce DENV-specific antibodies [25,26]. Previous studies have shown that most DENV-specific MBC produce serotype cross-reactive and poorly neutralizing antibodies. A small fraction of DENV-specific MBC encodes for serotype-specific and strongly neutralizing antibodies [[27], [28], [29], [30]]. Most strongly neutralizing antibodies bind to complex conformational epitopes of the viral envelope (E) protein that are efficiently displayed on intact virions but not on E protein monomers [25,[31], [32], [33], [34]]. The polyclonal LLPC-derived serum antibody response contains both poorly neutralizing cross-reactive antibodies and a small fraction of serotype-specific strongly neutralizing antibodies [25,28,31].

In contrast to the MBC and LLPC responses, the early plasmablast response to primary DENV infection is less clear. Several groups have characterized the early plasmablast response following secondary DENV infections [19,20,35,36]. DENV-specific plasmablasts accounted for 30–50% of circulating B cells during an ongoing secondary infection [19,35,37,38]. In secondary infections, the magnitude of the plasmablast response has been positively correlated with disease severity [35]. In secondary dengue the vast majority of the plasmablast pool is cross-reactive [38,39] and not maintained into convalescence [40]. Here we aim to characterize plasmblablast kinetics, magnitude, and specificity in primary DENV infection.

To longitudinally define the functionality of the plasmablast, MBC, and serum compartments following a primary DENV infection, we analyzed DENV-specific responses in each of these compartments in the same individuals following infection with a DENV2 challenge virus [41]. In this controlled infection model, a primary DENV2 infection stimulated a rapid plasmablast response wherein a large fraction of these cells produced serotype-specific antibodies to DENV2. The dominance of DENV2 serotype-specific antibodies was also observed at late convalescence (6 months after infection) in the MBC and LLPC compartments. Overall, the properties of antigen-specific antibodies in the early phase are also reflected in the late convalescent phase.

## 2 Materials and methods

### 2.1 Study participants

For this study, subjects received 10^3^ focus-forming units of the partially attenuated DENV2 human infection virus rDEN2Δ30, Tonga/74 strain by subcutaneous injection and followed for six months. Ten subjects were participants in a stand-alone trial for the safety and immunogenicity of rDEN2Δ30/Tonga/74 (CIR286, Clinicaltrials.gov NCT01931176, ref. [42] and nine were participants in the placebo/challenge arm of a Phase II vaccine/challenge clinical trial to test the safety and immunogenicity of the tetravalent live attenuated dengue vaccine TV003 (CIR287, Clinicaltrials.gov NCT02021968) followed by rDEN2Δ30/Tonga/74 challenge [41]. All subjects were serologically confirmed as flavivirus-naïve at the time of rDEN2Δ30 challenge, as described previously [41]. Studies were approved by the Institutional Review Boards at the University of Vermont and/or Johns Hopkins University. Informed consent was obtained in accordance with federal and international regulations (21CFR50 and ICHE6). External independent monitoring was performed by the National Institute of Allergy and Infectious Diseases Data Safety Monitoring Board every 6 months.

### 2.2 Clinical procedures and collection of samples

At study visits, blood was collected by venipuncture into serum separator tubes for analysis of viremia and serology, and into EDTA tubes for isolation of peripheral blood mononuclear cells (PBMC). Serum was isolated and frozen at –20 °C until use. PBMC were isolated by Ficoll-paque density gradient separation, counted and frozen in 10% DMSO/40% fetal bovine serum (FBS) in culture medium and cryopreserved in liquid nitrogen vapor phase.

### 2.3 Virus quantification and serologic response in subjects

Serum samples collected at follow-up visits every other day through day 16 were tested for infectious virus by direct titration on Vero-81 cells (American Type Culture Collection; CCL-81, RRID: CVCL_0059), and foci were detected with serotype-specific monoclonal antibodies as described [43]. Neutralizing antibody responses against DENV1 (WestPac/74), DENV2 (New Guinea C and Tonga/74), DENV3 (Slemen/78), and DENV4 (Dominica/81) were measured by focus reduction neutralization test (FRNT), using the lowest serum dilution that gave a 50% reduction in viral foci (FRNT_50_) as described [43]. Unless otherwise specified, these viruses were used for determination of DENV2 serotype-specificity and cross-reactivity of antibodies derived from the plasmablasts, MBC, and serum in ELISA or neutralization assays.

## 3 Analysis of plasmablasts and memory B cells

### 3.1 Plasmablast phenotyping

PBMC at days 0, 4, 8,14, 21, 28, 56, 90, 150 and 180 after rDEN2Δ30 challenge were stained with: anti-CD19 (clone HIB19, PE-Dazzle 594, BioLegend), anti-CD20 (2H7, PE-Cy7, BioLegend), anti-CD27 (O323, Brilliant Violet 510, BioLegend), anti-CD38 (HIT2, AlexaFluor 647, BioLegend), anti-CD3 (UCHT1, FITC, BioLegend), and anti-CD14 (HCD14, FITC, BioLegend). Plasmablasts were phenotyped by gating for CD3^–^CD14^–^CD19^ + ^CD20^–^CD27^ + ^CD38^hi^ live blasting cells. Data was collected on a BD LSR II (QC'd daily and compensation set with compensation beads (BD Biosciences) and data were analyzed with FlowJo v10 (TreeStar).

### 3.2 Plasmablast sorting

PBMC at Days 8, 14, and 21 after rDEN2Δ30 challenge were stained with: anti-CD19 (HIB19, Brilliant Violet 421, BioLegend), anti-CD20 (2H7, PE-Cy7, BioLegend or L27, PerCP-Cy5.5, BD Biosciences), anti-CD27 (O323, Brilliant Violet 510, BioLegend), anti-CD38 (HIT2, Alexa647, BioLegend), anti-IgA (IS11-8E10, FITC, Miltenyi Biotec), anti-IgM (MHM-88, FITC, BioLegend), anti-CD3 (UCHT1, FITC, BioLegend), and anti-CD14 (HCD14, FITC, BioLegend). Sorting for IgG+ plasmablasts (one cell per well into 96-well plates) was performed by fluorescence–activated cell sorting (FACS) for CD3^–^CD14^–^IgA^–^IgM^–^CD19^ + ^CD20^low/–^CD27^ + ^CD38^hi^ cells using a BD FACSJazz and BD FACS Diva software. Cells were single-cell sorted into 96-well PCR plates containing lysis buffer (10 mM Tris-HCl, pH 7.6), with 2 mM dNTPs (New England Biolabs), 5 μM oligo(dT)20VN, and 1 unit/μl of RiboLock RNase Inhibitor (ThermoFisher Scientific). Single-cell sorted plates were stored at −80 °C until use for reverse transcription (RT).

Reverse transcription, PCR, barcode assignment, sequence assembly, *V(D)J* assignment, and identification of mutations were performed as described previously [44,45] with the following modifications: biotinylated oligo(dT) was used for reverse transcription, cDNA was extracted using Streptavidin C1 beads (Life Technologies), DNA concentrations were determined using qPCR (KAPA SYBR® FAST qPCR Kit for Titanium, Kapabiosystems), and a minimum coverage of 10 reads was required from each chain assembly to be included in the sequence repertoires. *V(D)J* assignment and mutation identification were performed using an implementation of SoDA [46]. Paired H– and L-chain sequences within each rDEN2Δ30 recipient's plasmablast repertoire were assigned to the same lineage if the H-chain V-gene usage, CDRH3 length, L-chain V-gene usage, and CDRL3 length were identical. H– and L-chain CDRs, as defined [47], were identified by aligning protein sequences to a hidden Markov model [48]. Sequences were further separated into putative lineages based on the degree of identity of the CDRH3 and CDRL3 sequences.

### 3.3 Selection, cloning of antibody genes and expression of monoclonal antibodies from plasmablasts

The different antibody lineages were ranked based on evidence for infection-driven expansion and convergence across subjects as described [49]. Briefly, the criteria used to rank the lineages were (1) the number of distinct plasmablast clones within each lineage indicative of expansion or biased response to the infection, (2) the number of mutations suggestive of affinity maturation, (3) overlap of lineages across the three subjects suggestive of convergent evolution, and (4) clonal lineages with apparent sequence similarity among the lineage's members, indicative of sharing common progenitors. From each of the 96 highest priority lineages, we selected one lineage member for recombinant expression and purification. Selected sequences were either from the plasmablast clone in the lineage with the highest identity to the consensus sequence of the lineage, or from the clone expressed by the greatest number of plasmablasts in the lineage.

The 96 antibody heavy and light chain gene pairs were cloned into mammalian expression vectors (Lake Pharma, Belmont, California). Each complete construct was confirmed by sequencing. A small scale (0.01 L) transient production was done in HEK293 cells that were seeded in a shake flask and expanded using chemically defined serum-free medium. For each antibody, both the heavy- and light-chain encoding DNA constructs were transiently co-transfected into cells. The cells were maintained as a batch-fed culture until the end of the production. The proteins were purified using Protein A purification. The conditioned media from the transient production run was harvested and clarified by centrifugation and filtration. The supernatant was loaded into a Protein A column pre-equilibrated with binding buffer. Washing buffer was passed through the column until the OD_280_ value (NanoDrop, ThermoScientific) was measured to be zero. The target protein was eluted with a low pH buffer; fractions were collected and filtered through a 0.2 μm membrane filter. The antibodies were in 200 mM HEPES, 100 mM NaCl, 50 mM NaOAc, pH 7.0 buffer. Protein concentration was calculated from the OD_280_ value and the calculated extinction coefficient. The average yield was 0.117 mg and the median yield was 0.08 mg. Ninety two of the 96 selected IGH/IGL pairs yielded sufficient protein for functional testing.

### 3.4 Memory B cell isolation and immortalization

Switched memory B cells were isolated from cryopreserved PBMC collected on day 180 following rDEN2Δ30 challenge. After thawing, PBMC viability was >80% as assessed by lack of DAPI staining (4, 6-diamidino-2-phenylindole, 5 μg per sample in PBS – analyzed by flow cytometry on a Miltenyi VYB auto-sampler). B cells were enriched by labeling PBMC with microbead-conjugated anti-CD22 antibody (Miltenyi, catalog no. 130–046-401) followed by magnetic field separation (Miltenyi MS columns) to an average purity of 85%. Switched memory B cells were purified from CD22-enriched B cells by labeling with anti-CD3 (UCHT1, FITC, Biolegend), anti-CD19 (HIB19, PE-Dazzle594, Biolegend), anti-CD27 (O323, PE-Cy7, Biolegend), and anti-IgM (MHM-88, PerCP-Cy5.5, BioLegend). Cells were sorted into complete culture medium (see below) by FACS from the live lymphocyte (FSC x SSC) gate for CD3^–^CD19^ + ^CD27^+^IgM^–^ cells on a BD FACSAriaIII using the BD FACSDiva software. Post-sort purity was ≥ 95%.

Purified memory B cells were immortalized with the introduction of BCL-6 and Bcl-xL using retroviral transduction as previously described [50]. Briefly, purified memory B cells were activated in a 24-well tissue culture-treated plate with 1 mL of Iscove's Modified Dulbecco's Medium (IMDM) (Gibco, ThermoFisher) containing 8% FBS (Atlanta Biologicals), penicillin (100 units/mL, Gibco), streptomycin (100 μg/mL, Gibco), 1 × 10^5^ irradiated (50 Gy) human CD40L (CD154)-expressing mouse L cell fibroblasts and 50 ng/mL recombinant human interleukin (rhIL)-21 (Peprotech) for 36–48 h at 37 °C, 5% CO_2_. Cells were then suspended in 0.25 mL of serum-free IMDM into 24-well non-tissue culture-treated plates coated with 30 ng/mL retronectin (Takara) and blocked with 2% human serum albumin. The suspension of activated B cells in serum-free medium was mixed with equal volume of retrovirus (in DMEM/F-12 with 8% FBS) and added to wells. Plates were centrifuged at room temperature for one hour at 700 × g and then incubated at 37 °C for 6 h to overnight. After incubation, cells were washed with complete IMDM and maintained with rhIL-21 and CD40L-L cells. At 7–10 days of culture, GFP expression was used as a marker of transduction and we observed subject-specific efficiencies of 25–70%. CD19^ +^ GFP^+^ B cells were then sorted at 50 cells/well into 96-well plates containing rhIL-21 and 1 × 10^4^ CD40L- L cells in complete IMDM using using a BD FACSAriaIII (using the “single cell sort mask” mode, which provides the highest possible accuracy (<4% variance) in dispensing cells. After culture for 2–3 weeks, polyclonal cultures were screened for DENV–reactive IgG by ELISA (see below). Recovery of polyclonal sorted cells was 100% and secretion of IgG into culture supernatant (mean of 110 ng/mL) was confirmed by capture ELISA (Capture Ab: AffiniPure goat anti-human IgG (Fcγ), Jackson Immunoresearch cat no. 109–005-008; detection: Peroxidase AffiniPure Goat Anti-Human IgG, Fcγ fragment-specific, Jackson Immunoresearch cat no.109–035-008).

### 3.5 mAb cloning from immortalized MBC

Selected DENV2-specific polyclonal cultures were single-cell sorted into monoclonal cultures (same mode as above) and grown on CD40L and rhIL-21 and then screened as above after three weeks. DENV-specific monoclonal cultures were further qualitatively tested for neutralization of DENV by incubation of virus with 30 μL of culture supernatants prior to infection of Vero cells and assessment of neutralizing activity by microneutralization assay (see below). From frozen cell pellets of monoclonal cultures we isolated RNA, performed nested PCR for *IGH* and *IGL* genes and sequenced using specific primers as described [51,52]. Sequences were input into IgBLAST (https://www.ncbi.nlm.nih.gov/igblast/) and compared to germline to determine variable heavy and light chain usage, *V(D)J* gene usage, somatic hypermutations, complementary determining region (CDR)3 sequence, and IgG subtype. We cloned the *VH* into human IgG1 (Genbank FJ475055) and *VL* into Ig-λ or Ig-κ expression vectors (FJ517647, FJ475056, respectively). Heavy- and light chain-encoding plasmids were verified by sequencing and co-transfected into HEK-293F cells (RRID: CVCL_6642) to produce mAbs as described [51,52].

### 3.6 rDENV epitope-mapping viruses

Recombinant viruses are constructed using a four-cDNA cloning strategy as described previously [53]. Briefly, the rDENV4/2 was created by introducing the envelope protein (E) domain III (EDIII) residues from DENV2 into the DENV4 A subclone (Sri Lanka 1992; GenBank KJ160504.1) and replacing E nucleotides 900 to 1179 with the corresponding nucleotides encoding variant DENV2 amino acids [53]. rDENV2/1 was also obtained in a similar fashion on a DENV2 S16803 infectious clone backbone. The human monoclonal antibody (hmAb) 1F4 is a strongly neutralizing serotype DENV1-specific antibody. Using cryo-electron microscopy, the footprint of hmAb 1F4 has been mapped to a quaternary epitope that spans the E monomers of a dimer near the envelope domain I/II hinge region. The DENV1 1F4 epitope was transplanted into the DENV2 (S16803) infectious clone by changing 30 amino acids in DENV2 to their counterparts in DENV1. The transplantation results in a virus, rDENV2/1, which maintains DENV2 serotype-specific hmAb 2D22 neutralization, and fully gains hmAb 1F4 neutralization. The full-length cDNAs were transcribed into genome-length RNAs using T7 polymerase, as previously described [[53], [54], [55]]. These transcripts were electroporated into C6/36 cells (RRID:CVCL_Z230), and cell culture supernatant containing viable virus was harvested. Virus was passaged twice on C6/36 cell monolayer cultures and stored at 80 °C. Passage 3 virus was used as our working stock.

### 3.7 ELISAs to detect virus, rE and rEDIII–binding antibodies

Non-chimeric viruses used in the ELISA screens and neutralization assays were grown in Vero-81 cells at 37 °C, as previously described [26]. Recombinant envelope protein (recE) (80% of E protein) from WHO reference DENV2 S16803 strain was produced in our laboratory or purchased from Hawaii Biotech, Inc. [56]. Recombinant EDIII protein from DENV2 was produced in our laboratory as described previously [57]. For detection of IgG reactive to whole DENV by ELISA, each batch of each serotype was titrated separately by ELISA using a fixed amount of highly-cross reactive serum sample (DT000) to achieve OD_405_ = 1.0 to normalize among serotypes and among batches in ELISA assays. Equivalent quantities of DENV virus was captured by plate-bound mouse anti-E mAb 4G2 overnight at 4 °C. Recombinant E proteins were directly coated (rE - 100 ng/well; rEDIII - 200 ng/well) on ELISA plates overnight at 4 °C [53]. Plates were blocked with 3% (vol/vol) normal goat serum (Gibco - ThermoFisher, USA), in Tris-buffered saline (TBS) containing 0.05% (vol/vol) Tween 20 (blocking buffer). Plasmablast-derived IgG1 mAbs were tested at a fixed concentration of 20 μg/ml. 50 μL of polyclonal supernatant from immortalized memory B cells was tested. Following four washes, alkaline-phosphatase conjugated secondary anti-human IgG antibodies were used to detect binding of primary antibodies with *p*-nitrophenyl phosphate substrate, and reaction colour changes at OD_405_ were quantified by spectrophotometry.

### 3.8 Determination of quality of neutralizing antibodies by depletion of immune serum with DENV viruses

To determine whether DENV2 serotype-specific or cross-reactive antibodies contribute to overall neutralization of immune serum, we performed antibody depletion studies as previously described [26]. The WHO reference strains for purified viral antigens are as follows: DENV1 (American genotype; strain West Pac74), DENV2 (Asian genotype; strain S16803), (provided by Robert Putnak, Walter Reed Army Institute of Research, Silver Spring, MD). These viral antigens were used for performing the depletions and also further used for confirmation ELISA and neutralization assays.

Purified DENV was adsorbed onto 4.5-μm-diameter polystyrene microspheres (Polysciences, Inc.) at a bead (μL) to ligand (μg) ratio of 5:2. Polystyrene beads were washed three times with 0.1 M borate buffer (pH 8.5) and incubated with the relevant purified DENV (DENV2 for homotypic depletions and DENV1 for heterotypic depletions) overnight at room temperature (RT). Control beads were incubated overnight with an equivalent amount of bovine serum albumin (BSA). The control and virus-adsorbed beads were blocked with BSA (10 mg/ml)–borate buffer for 30 min at RT three times and washed four times with phosphate-buffered saline (PBS). DENV2 immune sera from the three selected subjects were depleted of virus-specific antibodies by incubating the samples with virus-adsorbed beads for 1 h at 37 °C with end-over-end mixing. Samples were subjected to at least three sequential rounds of depletions before successful removal of the respective antibodies was confirmed by ELISA. The ability of the depleted samples to neutralize viruses of all of the four serotypes was tested after the confirmation ELISA. Neutralization capacity of the depleted DENV2 immune sera were measured using a flow cytometry-based neutralization assay with U937-DC-SIGN cells as previously described [31].

### 3.9 Vero microneutralization assays

We adapted the FRNT as previously described [53] to a 96-well format [58]. 96-well plates were coated with 2 × 10^4^ Vero-81 cells/well and incubated at 37 °C for 24 h. Neutralization titers were determined by three-fold serial dilutions of sera mixed with 50–100 focus forming units per well in DMEM/F-12 supplemented with 2% FBS. Neutralization activity of mAbs was tested similarly over a range of 1-1000 ng/mL. The antibody-virus mixtures were incubated at 37 °C for 1 h before transferring to the 96-well plates containing confluent Vero-81 monolayers. Following an additional 1 -h incubation at 37 °C, the monolayers were overlaid with Opti-MEM (Gibco, ThermoFisher) containing 2% FBS and 1% (wt/vol) carboxymethyl cellulose (Sigma). Infected plates were incubated for 2 days at 37 °C with 5% CO_2^,^_ followed by fixation with 50 μl 4% paraformaldehyde for each well. Fixed cells were then permeabilized and blocked with non-fat dried milk in permeabilization buffer. Then the plates were incubated with a mix of 4G2 and 2H2 primary antibodies for an hour at 37 °C, washed, followed by secondary horseradish peroxidase (HRP)- conjugated anti-mouse IgG (KPL) for an hour at 37 °C, washed again, and developed with True Blue peroxidase substrate (KPL). The reaction was stopped with water and the plates were air dried before counting the foci on an Immunospot® S6 analyzer (Cellular Technology Limited) using the Immunospot® double count software. We calculated IC_50_ values by using the sigmoidal dose response (variable slope) equation of Prism 6 (Graph Pad Software). Reported values were required to have an R^2^ > 0.75, a hill slope > 0.5, and an IC_50_ within the range of the dilutions.

## 4 Results

We utilized a human DENV infection model to analyze the B cell and serum antibody responses to DENV infection. The human infection model is based on challenging subjects with a partially attenuated strain of DENV2 (rDEN2Δ30/Tonga/74). A complete description of the virologic and clinical features of this model has been published [41,42]. Healthy, flavivirus-naïve subjects infected with rDEN2Δ30 were followed over a six-month period (Fig. S1).

### 4.1 Plasmablast response to rDEN2Δ30 in flavivirus-naïve subjects

**Fig. 1:**
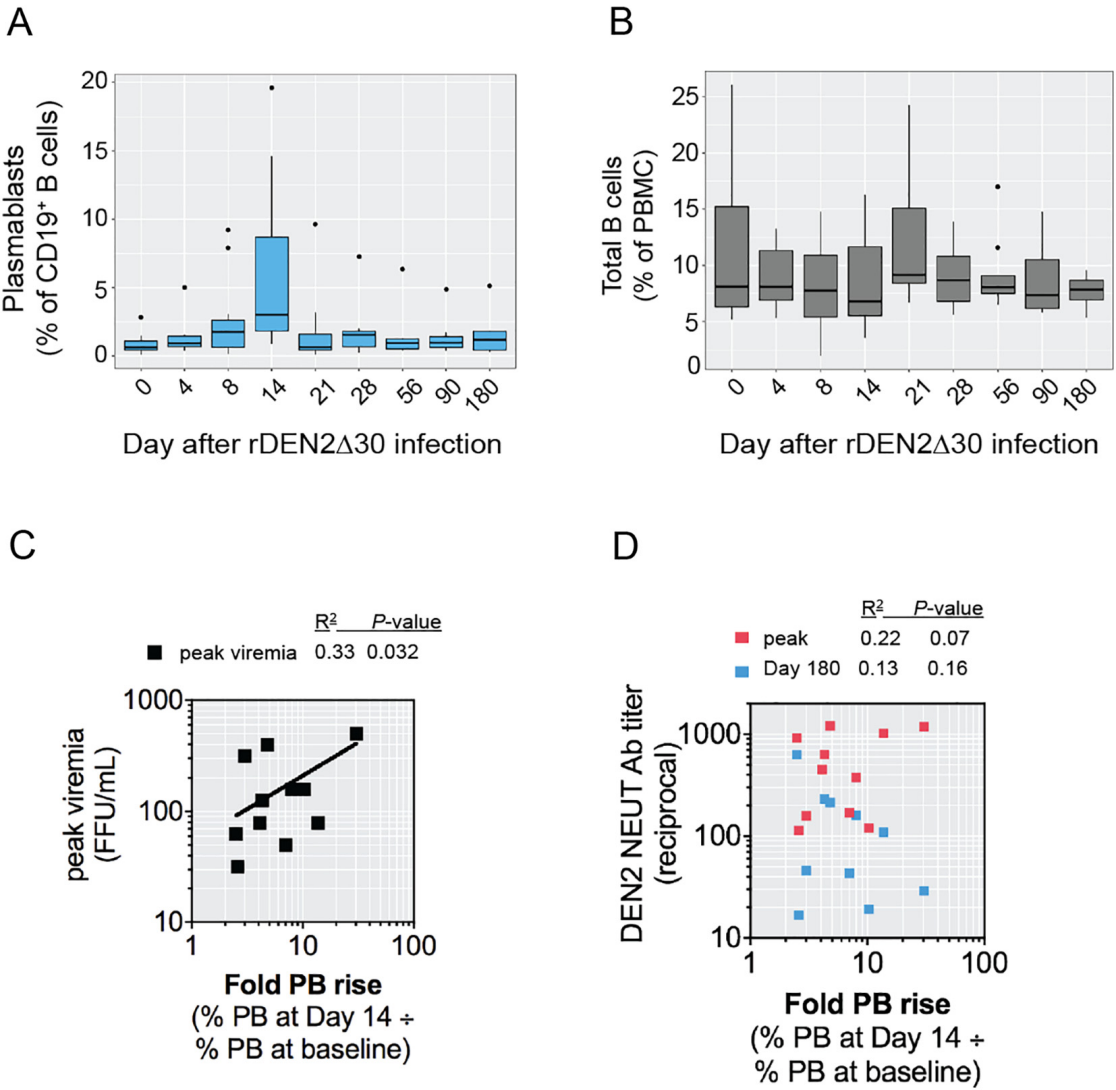
Kinetics of the plasmablast response to primary DENV2 infection. Flow cytometric analysis of (**A**) CD3^–^CD19^ + ^CD20^low/^^–^CD27^ + ^CD38^hi^ plasmablasts and (**B**) total CD19^+^ B cells in PBMC from subjects infected with rDEN2Δ30. Box and whiskers plots (Tukey) are shown (n = 12). (**C,D**) The fold-rise in plasmablasts on Day 14 versus baseline was plotted against (**C**) peak DENV2 viral load and (D) DENV2 neutralizing antibodies measured at peak after infection (Days 28–56) or at Day 180 after infection. Pearson's R-squared correlation coefficients and *P*-values are shown.

We first focused on plasmablasts, a population of antibody-secreting B cells that transiently increases in the blood early after viral infections [13] including DENV [19,20,35,36]. To determine whether a plasmablast increase was induced in the primary DENV2 infection model, we assessed the frequencies of CD19^ + ^CD20^–^CD27^ + ^CD38^hi^ cells in cryopreserved PBMC of twelve subjects infected with rDEN2Δ30. Plasmablast levels increased beginning at day 8 post-infection, peaked at day 14 and waned by day 21 (Fig. 1A, Fig. S2A), ranging 1.5–20% of the total CD19^+^ B cell population among subjects, and expanding on average 8.7–fold (range 2.5–30-fold) from day 0 to peak response (Fig. S2A). We assessed the percentage of peripheral B cells in total PBMC to determine whether change in plasmablast percentage was a function of total B cells. Generally, the percentage of peripheral B cells remained stable before and throughout the 6-month period following the infection (Fig. 1B, Fig. S2B and S3).

In people exposed to natural DENV infections, plasmablast levels have been correlated with peak viral titer [19,59]. Similarly, in this challenge model, the peak viral load was positively correlated with the plasmablast increase (Fig. 1C). We observed a weak correlation between changes in the plasmablast levels and peak serum neutralizing antibody titer (Fig. 1D), although the degree of correlation did not reach significance (*P* =0^ ^.07).

### 4.2 The plasmablast response to primary DENV2 infection is clonally expanded, and exhibits convergent evolution across subjects

**Fig. 2:**
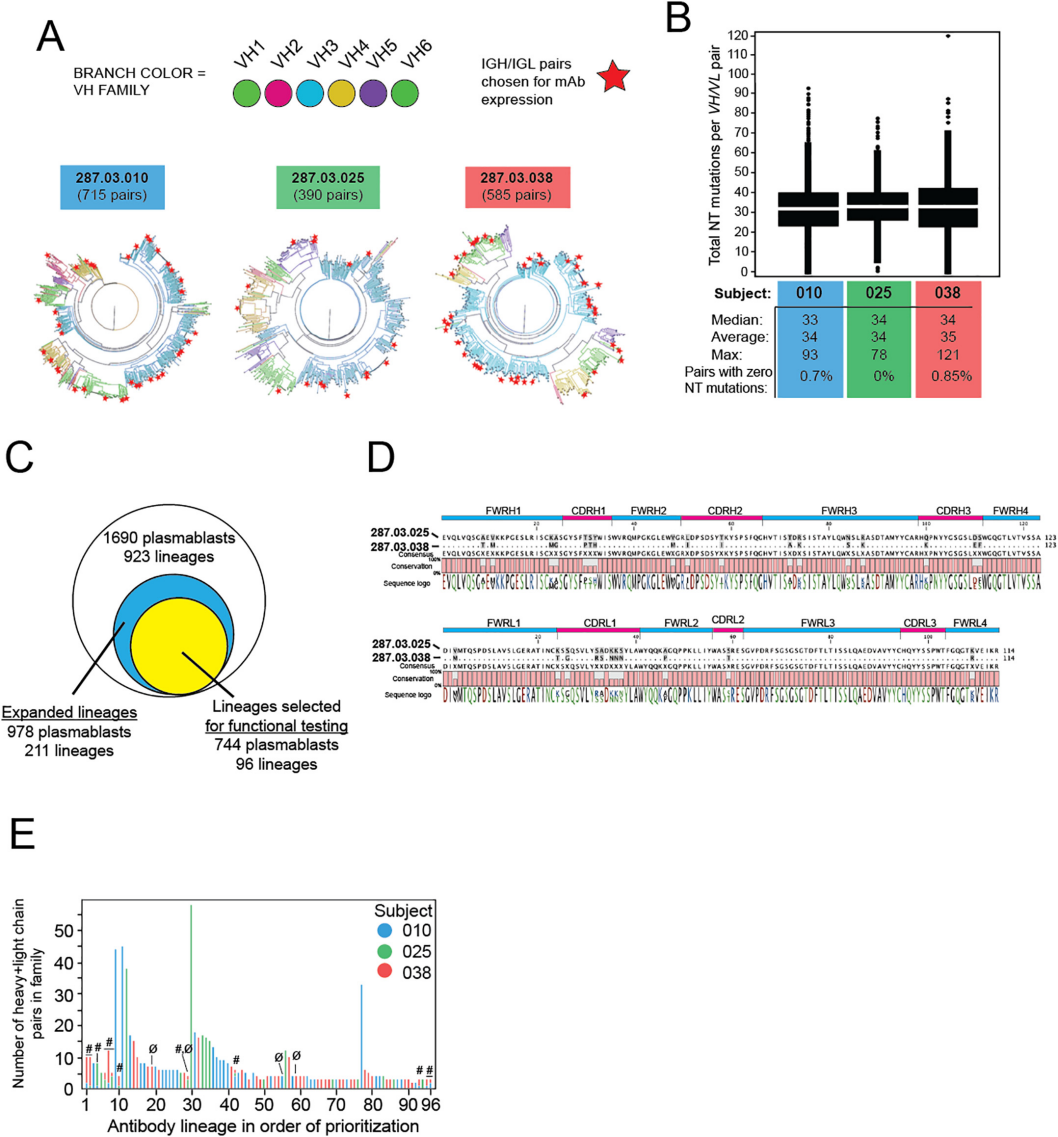
Evidence of somatic hypermutation and diversity in the plasmablast response to rDEN2Δ30. Plasmablasts from the peak response time points from three rDEN2Δ30 recipients were single-cell sorted and *IGH/IGL* sequencing was performed. Natively paired *IGH/IGL* sequences were compared to germline sequences and (**A**) phylograms were constructed to visualize *IGH/IGL* diversity. The distance from center is proportional to paired *VH/VL* somatic hypermutation compared to germline. *VH* gene usage is shown as colored branches. The number of unique plasmablasts sequenced from each donor is indicated. Stars indicate antibody sequence *VH/VL* pairs selected for recombinant IgG1 expression. (**B**) Median, average, and maximal values for sum of nucleotide mutations across paired *VH* and *VL* regions in all plasmablasts is shown for each subject. (**C**) Venn diagram of the total number of plasmablasts sequenced (with corresponding number of lineages), the number of cells contained within lineages showing evidence of expansion (i.e. lineages represented by at least 2 cells), and the number of cells within the expanded lineages that were selected (n = 96), and from which mAbs were recombinantly expressed. Note that the expressed mAbs represented 76% of the cells from lineages with evidence of expansion and 43% of the overall sequenced plasmablast repertoire. (**D**) High amino acid sequence identity across *IGH* and *IGL* variable regions from single plasmablasts from two different subjects challenged with rDEN2Δ30. Gray shading in aligned sequence indicates mismatch. Sequence logo coloring: green, polar/neutral; black, nonpolar/neutral; red, polar/acidic; blue, polar/basic. (**E**) Based on sequence and repertoire features (see methods), 96 lineages were prioritized for generation of an antibody screening library. A single antibody clone from the first 96 of these lineages was selected for recombinant IgG expression, including 12 pairs that were convergent (#) across two or more subjects (note the different colors representing different subjects in stacked bars). Four mAbs did not express as protein in sufficient quantity (noted with Ø), leaving 92 mAbs (including 11 convergent) for functional testing. (For interpretation of the references to colour in this figure legend, the reader is referred to the web version of this article.)

For three individuals across a range of rDEN2Δ30 viremia and plasmablast dynamics (Table 1) we performed in-depth longitudinal assessment of plasmablasts, MBC and serum antibodies in the same subjects over time. To characterize the plasmablast antibody repertoire, plasmablasts from each individual were single-cell sorted into 96-well plates on the basis of cell-surface staining for CD19^ + ^CD20^low/^^–^CD27^ + ^CD38^hi^ IgA^–^IgM^–^. A DNA barcoding method was utilized to obtain the paired heavy- and light-chain antibody sequences from a total of 1690 individual cells from the three subjects (Table S1) [44,60]. Phylogenetic trees of the plasmablast repertoires from the subjects were generated based on sequence homology of the paired heavy- and light-chain sequences (colored branches indicate heavy chain variable gene usage, Fig. 2A). Only 0.6% of these cells expressed naïve, unmutated antibodies (data not shown). The remaining plasmablasts exhibited an average rate of 34 nucleotide mutations per VH and VL pair (Fig. 2B), resulting in an average of 21 amino acid replacements across VH and VL per cell per subject (not shown).

**Table 1:** Viremia and plasmablasts in three rDEN2Δ30 recipients.

Subject	DENV2 Viremia			DENV2-induced plasmablasts					
	Onset (day)	Peak titer (log_10_ FFU/mL)	Day of peak	% Baseline	% at Day 14	Fold-rise (D14/Baseline)	Day of peak	% at peak	Fold-rise (Peak/Baseline)
010	8	1.5	10	0.5	1.3	2.6	21	2.7	5.4
025	6	2.1	6	1.5	9.6	6.5	14	9.6	6.5
038	4	2.9	4	0.3	3.1	10.3	14	3.1	10.3

Examples of clonally expanded plasmablast lineages were observed in all three subjects. The 1690 *IGH/IGL* pair sequences represented 923 lineages based on nucleotide sequence (clonal) similarity (Fig. 2C). A quarter of the lineages (211 of 923) were represented by at least two plasmablasts and were considered to have undergone proliferative expansion (i.e. *in vivo* selection). While the majority of blood plasmablasts sequenced (978/1690, 58% of total) belonged to expanded lineages, the remainder of the cells (712/1690, 42% of total) belonged to lineages comprising only one cell (singletons) (Table S2). Of the expanded lineages, we identified 48 lineages exhibiting convergent evolution. Members of convergent lineages shared germline *IGHV/IGLV* genes, CDRH3 and CDRL3 lengths, and exhibited ≥75% identity across the mature VH or VL peptide. An example of these convergent IGH/IGL pairs is shown in Fig. 2D. Taken together, these data show that primary DENV2 infection elicited a diverse pool of plasmablasts exhibiting clonal expansion, somatic hypermutation, and population of convergent lineages shared among subjects.

### 4.3 Binding properties of plasmablast-derived antibodies

**Fig. 3:**
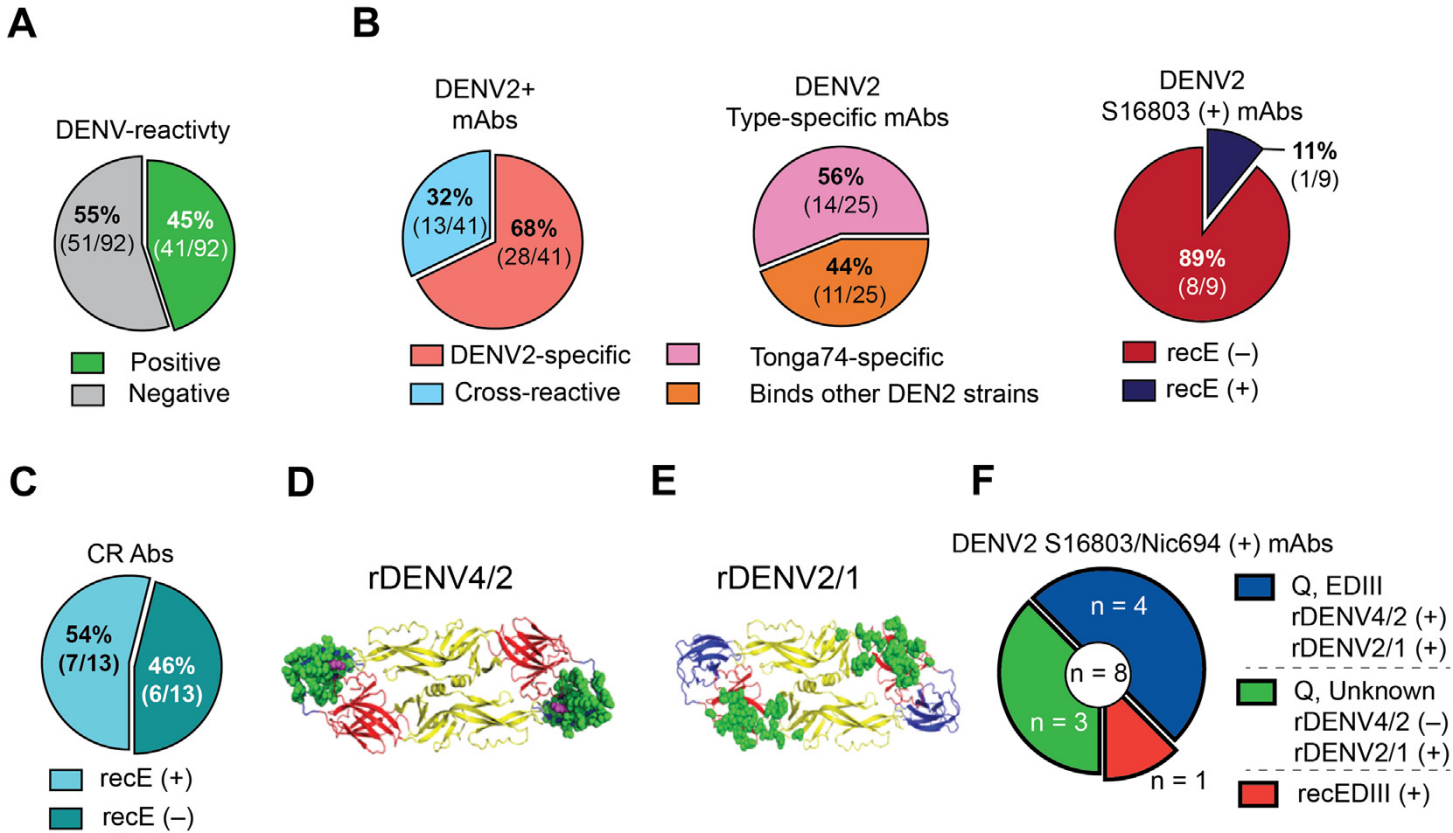
Binding properties of plasmablast-derived antibodies. (**A**) Fraction of plasmablast-derived DENV reactive hits and non-reactive antibodies and proportion of DENV2 serotype-specific and cross-reactive binders are shown. (**B**) Serotype-specificity of DENV2-positive mAbs (left) and indicated DENV2 strain-specificities (middle). Of those mAbs that bound to the WHO reference S16803 strain, the proportion that bound to S16803-derived recE are shown (right). (**C**). Proportion of cross-reactive mAbs which bind to recE are depicted. (**D**) Structural model of DENV4 E protein dimer, with swapped residues from EDIII of DENV2 (Nicaragua-694) colored in green to represent the rDENV4/2 (DENV2 EDIII virus) E glycoprotein and the DENV2 serotype-specific mAb 2D22 escape mutant residues highlighted in magenta. (**E**) Structural model of DENV2 (S16803) E protein dimer with DENV1-derived EDI and EI/II hinge residues swapped colored in green to represent the rDENV2/1 virus E glycoprotein. (**F**) Eight DENV2-specific mAbs binding to S16803 and Nicaragua-694 DENV2 viruses were tested for binding to recombinant EDIII (derived from S16803) as well as rDENV4/2 and rDENV2/1 chimeric viruses. The number and proportion of mAbs that bound to the indicated antigens is shown. Q: quaternary epitope-targeting mAbs. (For interpretation of the references to colour in this figure legend, the reader is referred to the web version of this article.)

To estimate the extent to which plasmablasts that expanded following primary DENV2 infection were able to recognize viral antigen, we selected 96 *IGH/IGL* pairs for recombinant expression and testing for DENV binding and neutralization (Fig. 2C). This number was chosen as a balance between adequate coverage of the expanded lineages (96 of 211, or 45%), resources, and compliance with a microplate-based cloning procedure. Within this group of expanded lineages selected for functional testing, we identified 12 convergent lineages (Fig. 2E, Table S3). 92 sequences yielded sufficient protein for further testing. These 92 lineages contained a total of 741 cells, which is 76% of the total number of cells in the 211 expanded lineages (978 cells) in the dataset (Table S2). Although all 92 antibodies were recombinantly expressed as IgG1, they were derived from native antibodies that were either IgG1 (83/92, 90%), IgG2 (3/92, 3%), or IgG3 isotypes (6/92, 7%). The final testing selection included mAbs from all three subjects, with 11 convergent sequences shared between at least two subjects (Fig. 2E). Forty five percent (41/92) of the recombinantly expressed antibodies bound to DENV2 Tonga/74 antigen (Fig. 3A). Of the DENV-binding antibodies, 32% were DENV serotype cross-reactive while 68% bound only to DENV2 (serotype-specific) (Fig. 3B). Of the eleven convergent sequences, six antibodies (55%) bound DENV2 antigen and one was neutralizing (Table S3). In addition, for each subject, we determined the percentage of plasmablasts in lineages producing DENV-binding antibodies among the total plasmablasts in lineages selected for recombinant expression (Table S2). In subjects 010, 025 and 038, the proportions of the plasmablasts in DENV-positive lineages were 32%, 70%, and 60% respectively. On the whole, the lineages we tested for binding that contained DENV2 reactive clones represented 54% of the plasmablasts among plasmablasts in all tested lineages.

Next, we compared the binding of the DENV2 serotype-specific mAbs to different strains of DENV2 represented by Tonga/74 (challenge strain, American genotype), New Guinea C (Asian genotype II), S16803 (Asian genotype I) and Nicaragua 694 (Asian/American genotype). Fifty six percent (14/25) of the tested DENV2 serotype-specific antibodies bound exclusively to Tonga/74 strain, while the remaining antibodies bound to at least two DENV2 strains (Fig. 3B). These results demonstrate that the early plasmablast response to primary DENV2 infection is a mixture of DENV serotype-specific and cross-reactive antibodies. Furthermore, nearly half the serotype-specific antibodies are strain-specific and recognize epitopes displayed on the DENV2 Tonga/74 virus but not other closely related DENV2 strains.

### 4.4 Mapping epitopes recognized by DENV-specific plasmablast-derived antibodies

We performed more detailed mapping studies with the DENV2 serotype-specific antibodies. Using recombinant proteins and chimeric virus reagents based on the DENV2 S16803 strain, we mapped the epitopes of 9 DENV2 serotype-specific antibodies that bound to S16803 (out of the 25 serotype-specific clones that bound to DENV2 Tonga/74). The majority (8/9) of these serotype-specific mAbs did not bind to recombinantly expressed E protein (recE) monomers derived from the same strain, indicating that quaternary structure E epitopes displayed on intact virions are likely targets of these antibodies (Fig. 3B). The one serotype-specific antibody that bound recE recognized a simple epitope on EDIII (data not shown). We also tested the 13 DENV cross-reactive antibodies for binding to recE. Fifty-four percent (7/13) of these antibodies bound to recE demonstrating that some cross-reactive antibodies bound to simple epitopes on monomeric E protein, while others recognized prM protein or E protein epitopes displayed only on intact virions (Fig. 3C).

To further map epitopes targeted by the DENV2 serotype-specific antibodies, we performed binding assays using recombinant chimeric DENV viruses with mutated E glycoproteins. rDENV4/2 is a DENV4 strain in which the entire DENV4 EDIII has been replaced with EDIII from DENV2-Nicaragua 694 (Fig. 3D). This recombinant virus has been used to map the binding of serotype-specific antibodies that target simple or complex epitopes centered on DENV2 EDIII [53]. rDENV2/1 is a DENV2 (S16803) virus in which residues in EDI and the hinge region between EDI and EDII have been mutated to display epitope for the DENV1 serotype-specific and neutralizing human antibody 1F4 (Fig. 3E). We, therefore, selected 8 mAbs that recognize both the S16803 and Nicaragua-694 strains for analysis. Four of the 8 mAbs directed at DENV2-specific quaternary epitopes (Fig. 3B) showed binding to rDENV4/2 and showed no appreciable loss of binding to the rDENV2/1 strain, establishing quaternary epitopes centered on DENV2 EDIII as the main target of binding (Fig. 3F, shown in blue). One mAb was DENV2 recE-reactive and targeted simple monomeric epitopes on recombinant EDIII (Fig. 3F, indicated in red). The remaining three mAbs bound to rDENV2/1 but not to rDENV4/2 indicating that residues on EDIII or the EDI/II hinge region were unlikely to be critical residues for binding (Fig. 3F, shown in green). Overall, these results demonstrate that most plasmablast–derived cross-reactive mAbs targeted simple epitopes on recE protein whereas the serotype-specific mAbs recognize quaternary structure epitopes centered on EDIII, including novel regions that do not map to known DENV2-specific mAb epitopes such as that of 2D22 [61].

### 4.5 Functional neutralization of DENV2 by plasmablast–derived mAbs

**Fig. 4:**
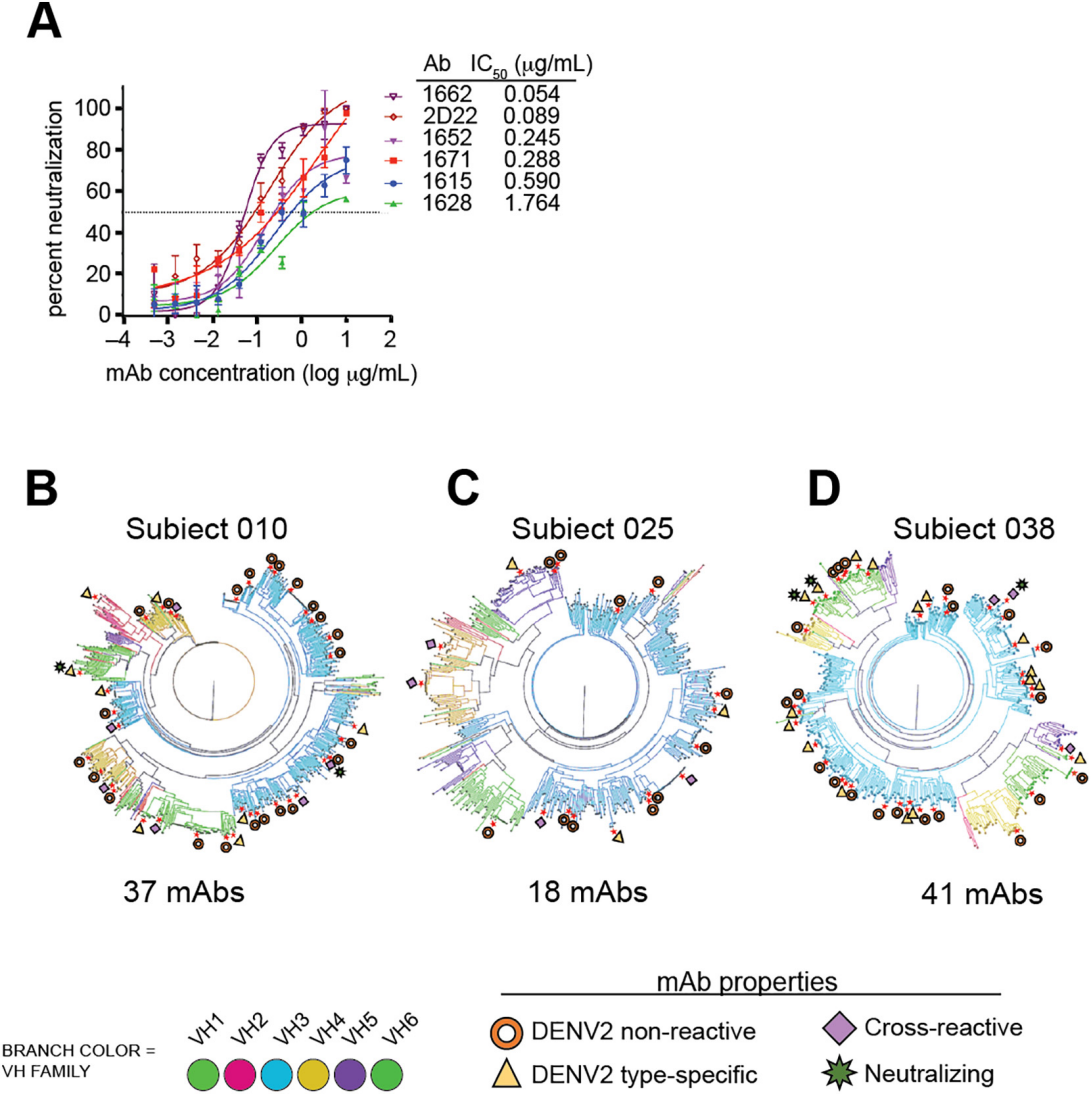
DENV2 neutralizing antibodies from plasmablasts. (**A**) Vero microneutralization assays were conducted with all the DENV2 reactive hits to determine the neutralization of DENV2 Tonga 74. Neutralization curves for five neutralizing antibodies are shown. Each point represents the mean neutralization value ± standard error of the mean from at least two experiments.,. (**B-D**) Phylogenetic trees depicting the properties of the antibodies tested in all the three subjects is shown.

We tested all DENV-binding mAbs in a DENV2 neutralization assay and identified five neutralizing mAbs, with potencies (assessed by IC_50_ concentrations) ranging 50–1700 ng/mL (Fig. 4A). Four of five were DENV2 serotype-specific (Table 2), including mAb 1662, with a potency that rivals 2D22, a strong DENV2-neutralizing mAb [31]. These five neutralizing mAbs were not DENV2 Tonga74 strain-specific. Four of five mAbs bound to all the DENV2 strains/genotypes tested in the study. Only one mAb (1615) did not bind to S16803 strain but bound to all other strains. Moreover, the three serotype-specific neutralizing mAbs that bound to epitopes on whole DENV2 virions and not to recE showed gain of binding to rDENV4/2 virus and did not lose binding to rDENV2/1. These results show that the three serotype-specific neutralizing mAbs targeted quaternary epitopes in the EDIII region. In Fig. 4B–D, we mapped all DENV2 serotype-specific, cross-reactive and neutralization hits from each of the three subjects to the phylogenic plasmablast trees but were unable to discern particular VH usage or clonal lineage characteristics predictive of functional activity.

**Table 2:** Detailed epitope mapping and functional characterization of DENV2 neutralizing mAbs derived from plasmablasts.

mAb	Subject	Binds DEN2 Tonga^a^	recE/Q^b^	rEDIII binding^c^	TS/CR^d^	NEUT_50_ (μg/mL)^e^
1662	010	+	Q	−	TS	0.054
1652	038	+	recE	+	CR	0.245
1671	038	+	Q	NB	TS	0.28
1615	010	+	ND	ND	TS	0.59
1628	038	+	Q	NB	TS	1.764

a Whole virus ELISA, “+” = O.D. ≥ 2-fold above blank.

b binding to recombinant E from DENV2 (recE). If positive, noted as “recE”. If negative, noted as quaternary (Q) epitope. ND, not determined as this antibody does not bind to the parental WHO S16803 strain from which recE is derived.

c binding to recombinant E domain III from DENV2 (rEDIII). NB, no binding; ND, not determined.

d whole virus ELISA. If bound to DENV2 only, noted as serotype-specific (TS); noted as cross-reactive (CR) if binding to DENV2 plus at least one other serotype found.

e microneutralization assay using Vero cells, NEUT50 is concentration of mAb required for ≥50% reduction in virus infection compared to negative control.

### 4.6 Memory B cells following DENV2 primary infection

**Fig. 5:**
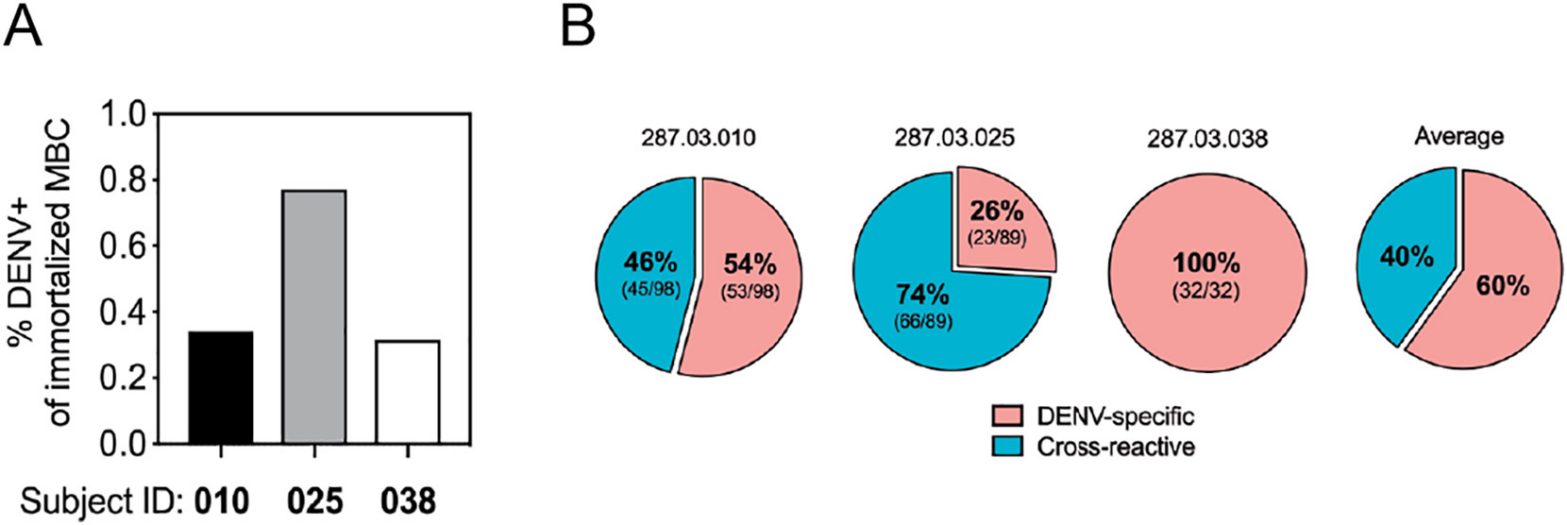
Elicitation of DENV-specific memory B cells by rDEN2Δ30 infection. (**A**) Total CD19^ + ^IgM^–^CD27^+^ switched MBC were isolated six months after rDEN2Δ30 infection in the same three subjects (287.03.010, 287.03.025, and 287.03.038) from which the plasmablast analyses were conducted. MBC from each subject were immortalized with a retrovirus encoding BCL6, Bcl-xL (6XL) and the transduction marker green fluorescent protein (GFP). 6XL-immortalized MBC were sorted by FACS for GFP and cultured at 50 cells/well; supernatants were screened for IgG binding to DENV2 Tonga/74. For each subject, frequency of DENV-reactive MBC was calculated from the number of positive wells divided by the total number of 6XL^+^ cells cultured and based on the presence of an average one positive clone yielding the positive signal per 50 cell input. (**B**) From each subject, DENV2-binding MBC were screened for reactivity to other serotypes by ELISA. The proportions (and numbers) of 219 MBC cultures that were DENV2 serotype-specific or cross-reactive with at least one other serotype (cross-reactive) are presented. The average proportions of serotype-specific versus cross-reactive cultures across the three subjects is also shown. (For interpretation of the references to colour in this figure legend, the reader is referred to the web version of this article.)

Our next objective was to evaluate the properties of memory B cells (MBC) six months after primary DENV2 infection in these three subjects. We utilized previously described genetic reprogramming approach based on retroviral transduction of the germinal center master regulator B cell lymphoma (BCL)-6 and the anti-apoptotic protein BCL-xL (together known as 6XL) to immortalize MBC from the three subjects [50]. To estimate the fraction of the MBC producing DENV-binding antibodies, we screened immunoglobulin-containing culture supernatants from 6XL-transduced cells by DENV capture ELISA (Fig. S4, Table S4) [26,31]. The frequencies of DENV-specific MBC were calculated using the number of positive cultures and the total number of transduced B cells sorted. We estimated an average 0.4% (219/52800) of total MBC to be DENV-specific, with the highest responder, subject 287.03.025, exhibiting a frequency of 0.74% (89 positive cultures out of 12,000 total 6XL^+^ cells plated) DENV2-reactive MBC followed by 287.03.010 with a frequency of 0.34% (98 positives out of 28,800 6XL^+^ cells), and 287.03.038 with a frequency of 0.28% (32 positives of 12,000 6XL^+^ cells) (Fig. 5A).

### 4.7 Binding specificity of MBC-derived antibodies

We screened the 219 DENV2- binding MBC culture supernatants from these three subjects to determine the extent to which these antibodies cross-reacted with other DENV serotypes. We found both serotype-specific MBC and cross-reactive MBC with bi-, tri-, and tetravalent reactivities. The serotype-specific fraction of the DENV2-reactive MBC response was donor-dependent; and across three subjects, an average of 60% of DENV2-reactive MBC was DENV2 serotype-specific and 40% was cross-reactive with DENV2 and at least one other serotype (Fig. 5B).

From one of the subjects (287.03.010) we isolated four DENV2-specific mAbs, one of which neutralizing, that represented four distinct lineages (Table S5). None of these paired IGH/IGL sequences was identified in the plasmablasts sampled from this subject. This lack of clonal overlap between the two B cell compartments has been observed in a prior study of secondary dengue [40]. Taken together, these data demonstrate the functional maintenance of serotype-specific humoral response in convalescence following primary DENV infection.

### 4.8 Quality of the convalescent polyclonal serum neutralizing antibodies following DENV2 infection

**Fig. 6:**
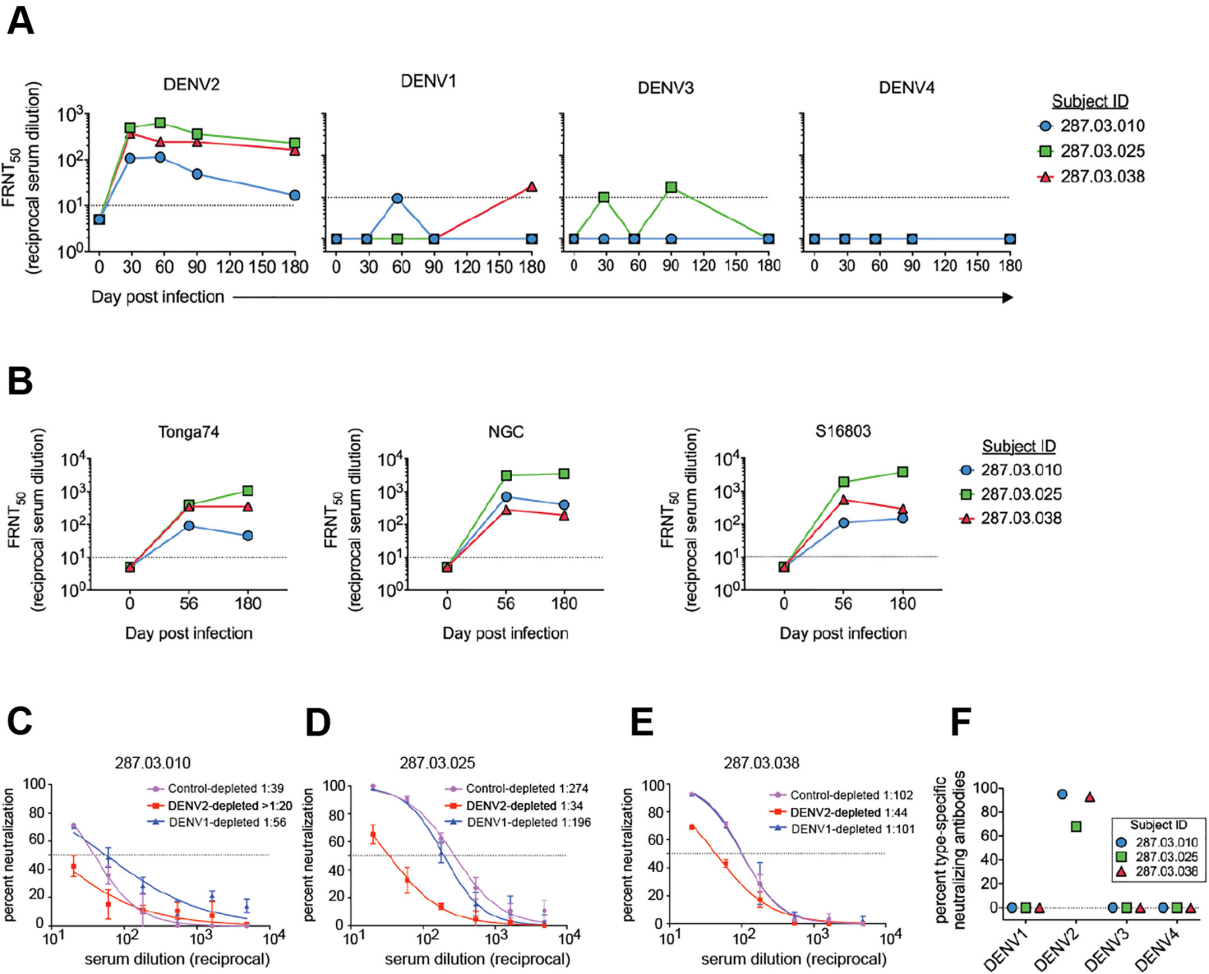
The properties of convalescent serum neutralizing antibodies following DENV2 infection. (**A**) 6 months after infection, sera from the three subjects were tested against DENV1–4 in a neutralization assay. (**B**) Sera from time points at day 0, 56, and 180 from the three subjects were tested for ability to neutralize DENV2 Tonga/74 (challenge strain, American genotype) and New Guinea C (Asian genotype II) in a microneutralization assay. For A and B, dotted lines indicate the lowest serum dilution factor used (1:10) in the neutralization assays and also the threshold above which a serum titer is considered positive. (**C-E**) 6 months post infection sera were depleted of antibodies binding to DENV2 antigen or DENV1 antigen. Control depletions were performed using bovine serum albumin as an antigen. Results presented here are from 2 technical replicates in one experiment. Neutralization curves are against DENV2 antigen for each subject. Each point in the neutralization curves represents the mean neutralization value from the two replicates, and the error bars depict the standard deviation. Dotted lines depict 50% neutralization. (**F**) All the three subjects develop DENV2 serotype-specific antibodies that mainly contribute to DENV2 neutralization.

Next, we characterized the properties of convalescent stage (6 months after infection) serum neutralizing antibodies. All three subjects developed neutralizing antibodies that neutralized DENV2 but not DENV1, 3 and 4 (Fig. 6A). The immune sera efficiently neutralized the infection DENV2 strain (Tonga/74) as well as two other strains belonging to different DENV2 genotypes (NGC and S16803) (Fig. 6B, Table S6). We performed antibody depletion studies to further define levels of DENV2 serotype-specific and cross-reactive neutralizing antibodies. Polystyrene beads coated with the homotypic (DENV2) or heterotypic (DENV1) serotypes were incubated to deplete specific populations of antibodies. Depletion of antibodies was confirmed by ELISA before using the samples in neutralization assays (data not shown). Depleting the Day 180 serum samples with the homotypic DENV2 antigen led to the removal of nearly all the DENV specific (DENV2 serotype-specific and cross reactive) antibodies in the samples as well as a significant loss of neutralizing activity. When only the DENV serotype cross-reactive antibodies were depleted in each sample (depletion with DENV1 antigen), there was minimal reduction in neutralization of DENV2 (Fig. 6C–E). On average ~89% of the serum neutralizing activity tracked with DENV2 serotype-specific antibodies (Fig. 6F).

**Fig. 7:**
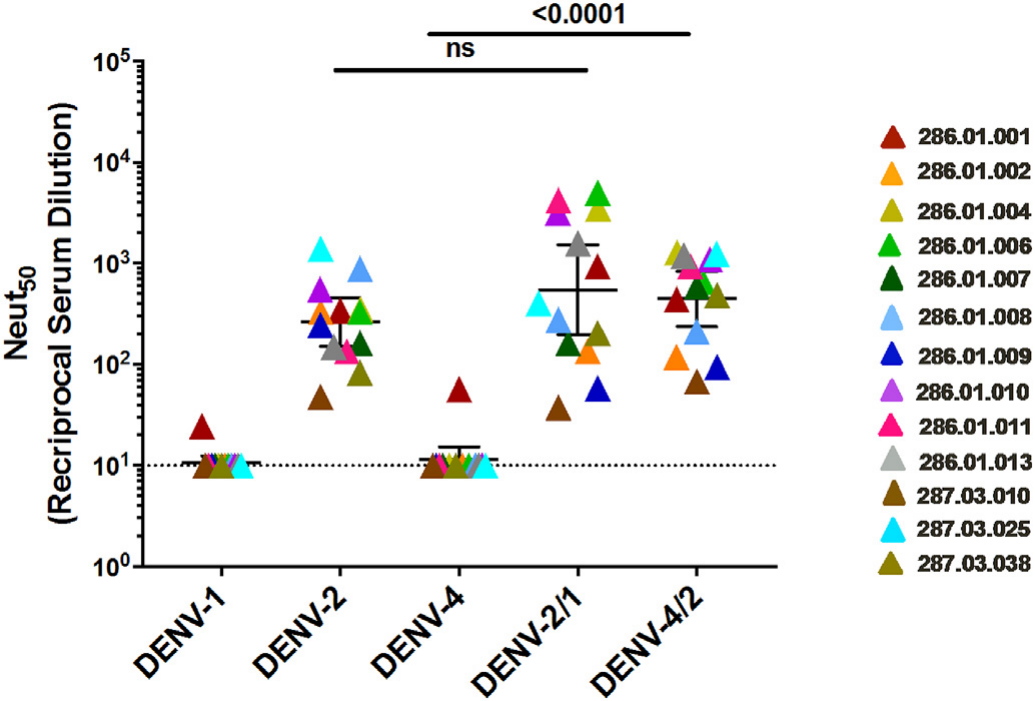
The DENV2 serotype-specific neutralizing antibodies target epitopes centered in the EDIII region. Neutralization assays were performed using a panel of polyclonal sera from subjects who received the Tonga 74 DENV2 strain using the rDENV4/2 and rDENV2/1 chimeric and wild type viruses. Each colour represents a single sample, and the neutralization against each virus (x-axis). Bars indicate the geometric mean titers and 95% confidence intervals of the grouped samples. *P*-values were calculated by Kruskal-Wallis followed by Dunn's post-hoc test.

To further characterize the epitopes targeted by the DENV2 serotype-specific neutralizing antibodies elicited by the rDEN2Δ30 infection, we performed neutralization assays using the rDENV2/1 and rDENV4/2 viruses. We also performed the assays with an additional panel of ten serum samples collected from individuals who received the rDEN2Δ30 challenge. Samples collected 180 days after infection were tested for neutralization capacity against the rDENV2/1 and rDENV4/2 and parental viruses (Fig. 7). All of the tested samples neutralized DENV2 well, and DENV1 and four poorly. The rDENV2/1 virus displaying the DENV1 1F4 epitope was neutralized at the same level as DENV2, demonstrating that the EDI region on DENV2 disrupted by the DENV1 1F4 epitope transplant were not a major target of neutralizing antibodies induced in people infected with the rDEN2Δ30. The neutralizing antibody response induced by rDEN2Δ30 was mainly directed to epitopes centered on EDIII because rDENV4/2 displaying EDIII from DENV2 was neutralized to the same level as the parental DENV2.

## 5 Discussion

Strong neutralizing antibodies play an essential role in viral clearance and thereby contribute to long-term protection in DENV infection [31]. However, the evolution of these rare neutralizing antibodies is poorly understood. Using a unique human DENV2 infection model, we have provided a longitudinal analysis of the continuum of the B cell response to primary DENV infection. Overall, we found that the functionality in terms of DENV2 serotype-specificity is conserved from the acute plasmablast response through the convalescent MBC and serological compartments (likely reflecting LLPC-derived antibodies). We propose that this framework to track the development of DENV-specific B cells, including those that produce serotype-specific antibodies, will be useful for evaluation of live attenuated DENV vaccine candidates, and to aid in deconvoluting the complex serology often found in natural infection.

During an acute secondary DENV infection, the plasmablast response can comprise a substantial fraction (36–95%) of the total CD19^+^ B cell response [19,35]. In our DENV2 primary infection model, we observed an average plasmablast peak of 6.0% of the total B cell population. Our results are similar to a recent study reporting a plasmablast peak of 2.5% of peripheral B cells among people exposed to Zika as a primary flavivirus infection [62]. In another study of 84 patients with laboratory-confirmed primary or secondary DENV infections, the magnitude of plasmablast response was substantially greater in secondary compared to primary infections [35]. These studies suggest that primary flavivirus infections elicit a lower plasmablast response compared to secondary infection. The higher response in secondary DENV infection may be due to a recall response upon encounter with similar DENV antigens. Another possibility arising from the association of the plasmablast response and viremia, shown here and elsewhere, suggests viral replication as a putative deterministic regulator of the B cell response [19]. It will be essential to determine whether low replication level of a tetravalent live attenuated dengue vaccine viruses compared to the rDEN2Δ30 challenge virus [41,63], predicts a modest plasmablast response. It is also possible, however, that the simultaneous administration of four viruses elicits a plasmablast response distinct from natural infection or primary challenge with rDEN2Δ30.

Plasmablast expansion to DENV2 infection may be the manifestation of multiple immune activation mechanisms acting on the B cell compartment. During acute DENV infection myeloid cells such as monocytes stain positive for the NS3 antigen which marks replicating DENV [64]. Activated CD14^+^CD16^+^ monocytes have been connected to plasmablast differentiation and proliferation [59]. Additionally, direct interaction between DENV and B cells without surface B cell receptor/immunoglobulin engagement could also lead to global activation of B cells. Serum neutralizing antibodies against DENV2 developed in all subjects following the transient plasmablast response, suggesting an antigen-specific activation of the B cell response. In line with this, nearly half the plasmablast lineages tested produced antibodies that bound to DENV2. Moreover, the paired *IGH/IGL* repertoire analysis of the plasmablast response revealed that over half of blood plasmablasts belong to lineages exhibiting evidence of clonal expansion. We also observed evidence of expanded convergent lineages among three subjects. The overall level of DENV2-binders identified, the clonality and shared lineages across subjects suggested the plasmablast proliferation was driven by cognate DENV antigens besides by purely bystander effects.

To estimate the degree of antigen-specificity of the plasmablast response, we characterized a panel of mAbs derived from a broad array of lineages comprising large, small, and convergent lineages. Using DENV binding ELISA we determined that 45% of the produced mAbs were DENV-specific. There are some limitations to our analysis. First, our approach did not fully cover the plasmablast landscape. The selected 92 lineages for antibody expression, though representing about 76% of the plasmablasts in all expanded lineages (741 of 978 cells), represented 44% of all plasmablasts sequenced. We reasoned that expanded lineages (representing 978 of 1690 total cells) would have a high likelihood of exhibiting DENV-specific activity when expressed. However, it is possible that some of the singleton lineages (n = 712) could be DENV-specific. Finally, only one antibody clone from each of these 92 lineages was selected for analysis. Continued effort is underway to characterize additional clones from these expanded and single plasmablast lineages to further refine our estimation of antigen-specificity in the plasmablast response. Since we tested mAbs from lineages representing 76% of those with evidence of expansion, and found that 45% of these bound DENV, our analysis suggests that at least 34% of the plasmablasts in lineages that expanded in response to primary rDEN2Δ30 infection are antigen-specific.

We further refined the specificity, antigenic regions, and functional properties of our mAb panel derived from primary rDEN2Δ30 infection. Screening for serotype cross-reactivity revealed that 68% of the plasmablast-derived mAbs recognized the infecting DENV2 serotype exclusively, and the remaining fraction showed cross-reactivity to DENV2 and at least one other serotype. Most of the serotype-specific antibodies targeted quaternary epitopes on intact virions, rather than simple epitopes on the viral E protein. This is contrary to a report on plasmablast-derived antibodies following secondary dengue infections where these antibodies were mainly cross-reactive and targeted simple E monomer epitopes and not complex epitopes [40]. We found DENV2-specific mAbs with functional activity similar to the mAb 2D22 isolated from natural infection [25,61,65,66] but also mAbs whose specificities could not be mapped to these epitopes or even to EDIII, suggesting the possibility that our panel of mAbs target uncharacterized DENV2 specificities. Five out of 41 DENV-reactive mAbs neutralized DENV2; indicating that neutralizing activity observed in serum may rely on only a small fraction of DENV-specific B cells despite the broad binding activity of the total repertoire. This finding and new DENV2-specific mAbs augment our repertoire of DENV2-specific reagents and may provide new insights to DENV2 neutralization mechanisms in addition to the few currently known DENV2-specific neutralizing mAbs [25,65,66]. We recently showed that DENV1 passaged by laboratory cell lines were structurally immature and hypersensitive to neutralization by human antibodies, particularly those that are heterotypic, as compared with DENV1 circulating in human subjects [67]. With the caveat that the same notion remains to be tested for DENV2, we posit that the type-specific DENV2 mAbs found here will neutralize circulating DENV2, while cross-reactive DENV2-binding antibodies may not.

It was striking that a high level of plasmablast-derived DENV-specific mAbs exhibited DENV2 strain specificity (towards DENV 2 Tonga/74). At the time of the epitope mapping work, only the recombinant antigens based on the WHO DENV2 strain (S16803) were available which directed our analyses to mAbs that bound S16803 strain. Based on recent publications regarding differential neutralization abilities of antibodies against different genotypes or strains within a serotype and failure of vaccines against genotypic variants [26,68], it is important to understand the epitopes targeted by these strain-specific antibodies and how this may change over time. However, this DENV2 strain-specificity was not seen in MBC (data not shown) (Fig. 6B), indicating that the memory response is broader than the plasmablast response. These observations suggest that it could be an intrinsic property of the plasmablast response to focus on the infecting DENV strain, while MBC may evolve to maintain broader specificity. Two limitations of our study are the small number of subjects in which we characterized the plasmablast and memory response to primary rDEN2Δ30 infection and that rDEN2Δ30 is a model of primary dengue infection. Nonetheless, Purtha and colleagues have reported on similar differences in the breadth of plasmablast and MBC compartments in a mouse model of West Nile virus infection [69].

The presence of antigen-specific MBC in circulation is one of the markers of long-term antiviral adaptive immunity [70]. To further interrogate the B cell response generated by primary DENV2 infection, we analyzed the DENV-specific repertoire of immortalized MBC 6-months post-infection. Across the three subjects we estimated an average of 0.4% of the immortalized MBC pool to be DENV-specific compared to 0.5–8.1% reported for secondary DENV infections by Appanna and colleagues [40]. Our estimate was also similar to a reported frequency (0.39%) of DENV-specific cells in the MBC pool following primary natural DENV infection and higher than the estimate from vaccination (0.08%) of immortalized MBC [71]. This level of antigen specificity in the MBC pool compared to natural infection supports the applicability of rDEN2∆30 for use as a physiologically relevant, but safe challenge virus [41,42].

In studies of natural infection, the DENV-specific MBC response has been characterized as overwhelmingly cross-reactive [25,28,66,71]. We find here that, similar to the plasmablast response, the MBC response to primary rDEN2Δ30 infection was biased towards DENV2 serotype-specificity. A possible explanation for this discrepancy may be the timepoints after infection at which MBC were studied;we immortalized MBC six months after infection, whereas previous studies utilized natural infection samples collected several years to decades after infection. Also, definitive determination of DENV exposure status in natural infection may be inexact. It is also possible that the MBC and serological response further converge after many years due to population of the LLPC compartment being replenished with MBC-derived clones over time [72].

Regarding the possibility of clonal overlap between the plasmablast and MBC compartments, we performed IGH/IGL sequencing on MBC from one of the subjects and identified four distinct lineages based on CDR3 sequences. We did not find these lineages in the plasmablast repertoire from the same subject. One possibility can be attributed to the limited number of unique MBC sequences isolated from that subject. Another possibility is that there might be little sharing of clones between the plasmablast and the MBC arms, implicating different sources of these clones during B cell development and differentiation. Indeed, Appanna and colleagues reported that plasmablasts during acute secondary DENV infection represent a small subset of the MBC pool [40]. Additionally recent work by Ellebedy and colleagues indicated that the activated B cell subset was phenotypically, transcriptionally, and functionally distinct from plasmablasts and contributes to the MBC pool to a greater extent than does the plasmablast pool [14].

The predominance of the serotype-specific response observed in the plasmablast repertoire was also corroborated in the serological data. Within 4 weeks following the infection, our subjects developed serum neutralizing antibodies against DENV2 but not DENV 1, 3 and 4, indicating that primary DENV infection in flavivirus-naïve subjects resulted in a homotypic neutralizing response focused on the infecting serotype. This is consistent with our work and that of others showing the importance of serotype-specific antibodies and their contribution in serum neutralization activity in natural infection [6,26,73]. This serological response was sustained into convalescence at 6 months post-infection despite the short-lived nature of the plasmablast population. The unique insight afforded by use of challenge model specimens was that the early B cell response to DENV infection contributes to the pool of long-lived plasma cells that produce serum antibody.

In summary, we defined the temporal and specificity characteristics of the early B cell response to primary DENV2 infection and showed that these early antibodies produced by acute stage plasmablasts functionally overlap with those produced in the long-term MBC and LLPC compartments. With the caveat that this study was done in a small number of subjects, our results help to further validate the rDEN2Δ30 human challenge model of primary DENV infection, as has also been done by analysis of the T cell response [74]. Next, our data linking plasmablast peak response with viremia may also provide a secondary means of indirectly monitoring viral replication in vivo, which will be important in the continued development of live attenuated dengue vaccines. In the context of both natural DENV infections and live attenuated vaccines, by profiling the acute stage plasmablast response, it may be possible to link viral replication with early plasmablasts and potentially identify protective responses at the cellular level. We propose that the framework presented here may be useful for its capacity to identify novel immune response patterns, many of which phenocopy events seen in natural infections, and to predict the quality and durability of long-term antibody responses to vaccination.

## Funding sources

This study was supported by the Bill and Melinda Gates Foundation (OPP110470), the National Institutes of Allergy and Infectious Disease (R01 AI107731–05 to A.DS, and T32AI055402 to H.T.), and the National Institutes of General Medical Sciences (P20GM125498 to B.D.K. and S.A.D.). The clinical trials from which samples were obtained were funded by NIAID Intramural contract HHSN272200900010C. The funders had no role in the study design; in the collection, analysis, and interpretation of data, in the writing of the report, or in the decision to submit the paper for publication.

## Conflict of interest

NN and DEE are employees of Atreca Inc. Other authors declare no conflict of interest.

## Author contributions

SAD, DEE, and AMD conceived and designed experiments. UKN, HAT, MJD, JS, BP and NN performed experiments. UNK, HAT, NN, DEE, RSB, AMD, and SAD analyzed the data. APD, SSW, KKP, and BDK provided specimens. UKN, HAT, AMD and SAD wrote the manuscript. All authors participated in the editorial process and approved the manuscript.

## Appendix A Supplementary data

Supplementary material Image 1

## References

[1] Guzman M.G., Gubler D.J., Izquierdo A., Martinez E., Halstead S.B. (2016). Dengue infection. Nat Rev Dis Primers.

[2] Guzman M.G., Dengue Harris E. (2015). Lancet..

[3] Bhatt S., Gething P.W., Brady O.J., Messina J.P., Farlow A.W., Moyes C.L. (2013). The global distribution and burden of dengue. Nature..

[4] Sabin A.B. (1952). Research on dengue during world war II. Am J Trop Med Hyg.

[5] Imrie A., Meeks J., Gurary A., Sukhbaatar M., Truong T.T., Cropp C.B. (2007). Antibody to dengue 1 detected more than 60 years after infection. Viral Immunol.

[6] Patel B., Longo P., Miley M.J., Montoya M., Harris E., de Silva A.M. (2017). Dissecting the human serum antibody response to secondary dengue virus infections. PLoS Negl Trop Dis.

[7] Halstead S.B., Nimmannitya S., Cohen S.N. (1970). Observations related to pathogenesis of dengue hemorrhagic fever. IV. Relation of disease severity to antibody response and virus recovered. Yale J Biol Med.

[8] Burke D.S., Nisalak A., Johnson D.E., Scott R.M. (1988). A prospective study of dengue infections in Bangkok. Am J Trop Med Hyg.

[9] Sangkawibha N., Rojanasuphot S., Ahandrik S., Viriyapongse S., Jatanasen S., Salitul V. (1984). Risk factors in dengue shock syndrome: a prospective epidemiologic study in Rayong, Thailand. I. The 1980 outbreak. Am J Epidemiol.

[10] Thein S., Aung M.M., Shwe T.N., Aye M., Zaw A., Aye K. (1997). Risk factors in dengue shock syndrome. Am J Trop Med Hyg.

[11] Guzman M.G., Alvarez M., Halstead S.B. (2013). Secondary infection as a risk factor for dengue hemorrhagic fever/dengue shock syndrome: an historical perspective and role of antibody-dependent enhancement of infection. Arch Virol.

[12] Katzelnick L.C., Gresh L., Halloran M.E., Mercado J.C., Kuan G., Gordon A. (2017). Antibody-dependent enhancement of severe dengue disease in humans. Science..

[13] Wrammert J., Smith K., Miller J., Langley W.A., Kokko K., Larsen C. (2008). Rapid cloning of high-affinity human monoclonal antibodies against influenza virus. Nature..

[14] Ellebedy A.H., Jackson K.J., Kissick H.T., Nakaya H.I., Davis C.W., Roskin K.M. (2016). Defining antigen-specific plasmablast and memory B cell subsets in human blood after viral infection or vaccination. Nat Immunol.

[15] Obermoser G., Presnell S., Domico K., Xu H., Wang Y., Anguiano E. (2013). Systems scale interactive exploration reveals quantitative and qualitative differences in response to influenza and pneumococcal vaccines. Immunity..

[16] Wrammert J., Koutsonanos D., Li G.M., Edupuganti S., Sui J., Morrissey M. (2011). Broadly cross-reactive antibodies dominate the human B cell response against 2009 pandemic H1N1 influenza virus infection. J Exp Med.

[17] Rahman A., Rashu R., Bhuiyan T.R., Chowdhury F., Khan A.I., Islam K. (2013). Antibody-secreting cell responses after Vibrio cholerae O1 infection and oral cholera vaccination in adults in Bangladesh. Clin Vaccine Immunol.

[18] McElroy A.K., Akondy R.S., Davis C.W., Ellebedy A.H., Mehta A.K., Kraft C.S. (2015). Human Ebola virus infection results in substantial immune activation. Proc Natl Acad Sci U S A.

[19] Wrammert J., Onlamoon N., Akondy R.S., Perng G.C., Polsrila K., Chandele A. (2012). Rapid and massive virus-specific plasmablast responses during acute dengue virus infection in humans. J Virol.

[20] Xu M., Hadinoto V., Appanna R., Joensson K., Toh Y.X., Balakrishnan T. (2012). Plasmablasts generated during repeated dengue infection are virus glycoprotein-specific and bind to multiple virus serotypes. J Immunol.

[21] Priyamvada L., Cho A., Onlamoon N., Zheng N.Y., Huang M., Kovalenkov Y. (2016). B cell responses during secondary Dengue virus infection are dominated by highly cross-reactive, memory-derived Plasmablasts. J Virol.

[22] Tangye S.G., Tarlinton D.M. (2009). Memory B cells: effectors of long-lived immune responses. Eur J Immunol.

[23] Schmidlin H., Diehl S.A., Blom B. (2009). New insights into the regulation of human B-cell differentiation. Trends Immunol.

[24] Smith T., Cunningham-Rundles C. (2018). Primary B-cell immunodeficiencies. Hum Immunol.

[25] Smith S.A., de Alwis A.R., Kose N., Jadi R.S., de Silva A.M., Crowe J.E. (2014). Isolation of dengue virus-specific memory B cells with live virus antigen from human subjects following natural infection reveals the presence of diverse novel functional groups of antibody clones. J Virol.

[26] Nivarthi U.K., Kose N., Sapparapu G., Widman D., Gallichotte E., Pfaff J.M. (2017). Mapping the human memory B cell and serum neutralizing antibody responses to Dengue virus serotype 4 infection and vaccination. J Virol.

[27] Schieffelin J.S., Costin J.M., Nicholson C.O., Orgeron N.M., Fontaine K.A., Isern S. (2010). Neutralizing and non-neutralizing monoclonal antibodies against dengue virus E protein derived from a naturally infected patient. Virol J.

[28] Beltramello M., Williams K.L., Simmons C.P., Macagno A., Simonelli L., Quyen N.T. (2010). The human immune response to Dengue virus is dominated by highly cross-reactive antibodies endowed with neutralizing and enhancing activity. Cell Host Microbe.

[29] Dejnirattisai W., Jumnainsong A., Onsirisakul N., Fitton P., Vasanawathana S., Limpitikul W. (2010). Cross-reacting antibodies enhance dengue virus infection in humans. Science.

[30] de Alwis R., Beltramello M., Messer W.B., Sukupolvi-Petty S., Wahala W.M., Kraus A. (2011). In-depth analysis of the antibody response of individuals exposed to primary dengue virus infection. PLoS Negl Trop Dis.

[31] de Alwis R., Smith S.A., Olivarez N.P., Messer W.B., Huynh J.P., Wahala W.M. (2012). Identification of human neutralizing antibodies that bind to complex epitopes on dengue virions. Proc Natl Acad Sci U S A.

[32] Teoh E.P., Kukkaro P., Teo E.W., Lim A.P., Tan T.T., Yip A. (2012). The structural basis for serotype-specific neutralization of dengue virus by a human antibody. Sci Transl Med.

[33] Friberg H., Jaiswal S., West K., O'Ketch M., Rothman A.L., Mathew A. (2012). Analysis of human monoclonal antibodies generated by dengue virus-specific memory B cells. Viral Immunol.

[34] Rouvinski A., Guardado-Calvo P., Barba-Spaeth G., Duquerroy S., Vaney M.C., Kikuti C.M. (2015). Recognition determinants of broadly neutralizing human antibodies against dengue viruses. Nature.

[35] Garcia-Bates T.M., Cordeiro M.T., Nascimento E.J., Smith A.P., Soares de Melo K.M., McBurney S.P. (2013). Association between magnitude of the virus-specific plasmablast response and disease severity in dengue patients. J Immunol.

[36] Dejnirattisai W., Wongwiwat W., Supasa S., Zhang X., Dai X., Rouvinski A. (2015). A new class of highly potent, broadly neutralizing antibodies isolated from viremic patients infected with dengue virus. Nat Immunol.

[37] Balakrishnan T., Bela-Ong D.B., Toh Y.X., Flamand M., Devi S., Koh M.B. (2011). Dengue virus activates polyreactive, natural IgG B cells after primary and secondary infection. PLoS One.

[38] Zompi S., Montoya M., Pohl M.O., Balmaseda A., Harris E. (2012). Dominant cross-reactive B cell response during secondary acute dengue virus infection in humans. PLoS Negl Trop Dis.

[39] Mathew A., West K., Kalayanarooj S., Gibbons R.V., Srikiatkhachorn A., Green S. (2011). B-cell responses during primary and secondary dengue virus infections in humans. J Infect Dis.

[40] Appanna R., Kg S., Xu M.H., Toh Y.X., Velumani S., Carbajo D. (2016). Plasmablasts during acute Dengue infection represent a small subset of a broader virus-specific memory B cell Pool. EBioMedicine.

[41] Kirkpatrick B.D., Whitehead S.S., Pierce K.K., Tibery C.M., Grier P.L., Hynes N.A. (2016). The live attenuated dengue vaccine TV003 elicits complete protection against dengue in a human challenge model. Sci Transl Med.

[42] Larsen C.P., Whitehead S.S., Durbin A.P. (2015). Dengue human infection models to advance dengue vaccine development. Vaccine.

[43] Durbin A.P., Karron R.A., Sun W., Vaughn D.W., Reynolds M.J., Perreault J.R. (2001). Attenuation and immunogenicity in humans of a live dengue virus type-4 vaccine candidate with a 30 nucleotide deletion in its 3′-untranslated region. Am J Trop Med Hyg.

[44] Tan Y.C., Kongpachith S., Blum L.K., Ju C.H., Lahey L.J., Lu D.R. (2014). Barcode-enabled sequencing of plasmablast antibody repertoires in rheumatoid arthritis. Arthritis Rheumatol.

[45] Tan Y.C., Blum L.K., Kongpachith S., Ju C.H., Cai X., Lindstrom T.M. (2014). High-throughput sequencing of natively paired antibody chains provides evidence for original antigenic sin shaping the antibody response to influenza vaccination. Clin Immunol.

[46] Volpe J.M., Cowell L.G., Kepler T.B. (2006). SoDA: implementation of a 3D alignment algorithm for inference of antigen receptor recombinations. Bioinformatics..

[47] Kabat E.A., Wu T.T., Reid-Miller M., Perry H.M., Gottesman K.S. (1987). Sequences of proteins of immunological interest: public health service, Bethesda, MD.

[48] Glanville J., Zhai W., Berka J., Telman D., Huerta G., Mehta G.R. (2009). Precise determination of the diversity of a combinatorial antibody library gives insight into the human immunoglobulin repertoire. Proc Natl Acad Sci U S A.

[49] Scally S.W., McLeod B., Bosch A., Miura K., Liang Q., Carroll S. (2017). Molecular definition of multiple sites of antibody inhibition of malaria transmission-blocking vaccine antigen Pfs25. Nat Commun.

[50] Kwakkenbos M.J., Diehl S.A., Yasuda E., Bakker A.Q., van Geelen C.M., Lukens M.V. (2010). Generation of stable monoclonal antibody-producing B cell receptor-positive human memory B cells by genetic programming. Nat Med.

[51] Ho I.Y., Bunker J.J., Erickson S.A., Neu K.E., Huang M., Cortese M. (2016). Refined protocol for generating monoclonal antibodies from single human and murine B cells. J Immunol Methods.

[52] Smith K., Garman L., Wrammert J., Zheng N.Y., Capra J.D., Ahmed R. (2009). Rapid generation of fully human monoclonal antibodies specific to a vaccinating antigen. Nat Protoc.

[53] Gallichotte E.N., Widman D.G., Yount B.L., Wahala W.M., Durbin A., Whitehead S. (2015). A new quaternary structure epitope on dengue virus serotype 2 is the target of durable type-specific neutralizing antibodies. MBio..

[54] Messer W.B., Yount B., Hacker K.E., Donaldson E.F., Huynh J.P., de Silva A.M. (2012). Development and characterization of a reverse genetic system for studying dengue virus serotype 3 strain variation and neutralization. PLoS Negl Trop Dis.

[55] Messer W.B., Yount B.L., Royal S.R., de Alwis R., Widman D.G., Smith S.A. (2016). Functional transplant of a Dengue virus serotype 3 (DENV3)-specific human monoclonal antibody epitope into DENV1. J Virol.

[56] Modis Y., Ogata S., Clements D., Harrison S.C. (2004). Structure of the dengue virus envelope protein after membrane fusion. Nature.

[57] Wahala W.M., Kraus A.A., Haymore L.B., Accavitti-Loper M.A., de Silva A.M. (2009). Dengue virus neutralization by human immune sera: role of envelope protein domain III-reactive antibody. Virology.

[58] Swanstrom J.A., Plante J.A., Plante K.S., Young E.F., McGowan E., Gallichotte E.N. (2016). Dengue virus envelope dimer epitope monoclonal antibodies isolated from Dengue patients are protective against Zika virus. MBio.

[59] Kwissa M., Nakaya H.I., Onlamoon N., Wrammert J., Villinger F., Perng G.C. (2014). Dengue virus infection induces expansion of a CD14(+)CD16(+) monocyte population that stimulates plasmablast differentiation. Cell Host Microbe.

[60] Lu D.R., Tan Y.C., Kongpachith S., Cai X., Stein E.A., Lindstrom T.M. (2014). Identifying functional anti-Staphylococcus aureus antibodies by sequencing antibody repertoires of patient plasmablasts. Clin Immunol.

[61] Gallichotte E.N., Baric T.J., Yount B.L., Widman D.G., Durbin A., Whitehead S. (2018). Human dengue virus serotype 2 neutralizing antibodies target two distinct quaternary epitopes. PLoS Pathog.

[62] Rogers T.F., Goodwin E.C., Briney B., Sok D., Beutler N., Strubel A. (2017). Zika virus activates de novo and cross-reactive memory B cell responses in dengue-experienced donors. Sci Immunol.

[63] Kirkpatrick B.D., Durbin A.P., Pierce K.K., Carmolli M.P., Tibery C.M., Grier P.L. (2015). Robust and balanced immune responses to all 4 Dengue virus serotypes following Administration of a Single Dose of a live attenuated tetravalent Dengue vaccine to healthy, Flavivirus-naive adults. J Infect Dis.

[64] Durbin A.P., Vargas M.J., Wanionek K., Hammond S.N., Gordon A., Rocha C. (2008). Phenotyping of peripheral blood mononuclear cells during acute dengue illness demonstrates infection and increased activation of monocytes in severe cases compared to classic dengue fever. Virology.

[65] Fibriansah G., Ibarra K.D., Ng T.S., Smith S.A., Tan J.L., Lim X.N. (2015). Dengue Virus. Cryo-EM structure of an antibody that neutralizes dengue virus type 2 by locking E protein dimers. Science.

[66] Smith S.A., Zhou Y., Olivarez N.P., Broadwater A.H., de Silva A.M., Crowe J.E. (2012). Persistence of circulating memory B cell clones with potential for dengue virus disease enhancement for decades following infection. J Virol.

[67] Raut R., Corbett K.S., Tennekoon R.N., Premawansa S., Wijewickrama A., Premawansa G. (2019). Dengue type 1 viruses circulating in humans are highly infectious and poorly neutralized by human antibodies. Proc Natl Acad Sci U S A.

[68] Gallichotte E.N., Baric T.J., Nivarthi U., Delacruz M.J., Graham R., Widman D.G. (2018). Genetic variation between Dengue virus type 4 strains impacts human antibody binding and neutralization. Cell Rep.

[69] Purtha W.E., Tedder T.F., Johnson S., Bhattacharya D., Diamond M.S. (2011). Memory B cells, but not long-lived plasma cells, possess antigen specificities for viral escape mutants. J Exp Med.

[70] Crotty S., Felgner P., Davies H., Glidewell J., Villarreal L., Ahmed R. (2003). Cutting edge: long-term B cell memory in humans after smallpox vaccination. J Immunol.

[71] Smith S.A., de Alwis R., Kose N., Durbin A.P., Whitehead S.S., de Silva A.M. (2013). Human monoclonal antibodies derived from memory B cells following live attenuated dengue virus vaccination or natural infection exhibit similar characteristics. J Infect Dis.

[72] Amanna I.J., Slifka M.K. (2010). Mechanisms that determine plasma cell lifespan and the duration of humoral immunity. Immunol Rev.

[73] Katzelnick L.C., Montoya M., Gresh L., Balmaseda A., Harris E. (2016). Neutralizing antibody titers against dengue virus correlate with protection from symptomatic infection in a longitudinal cohort. Proc Natl Acad Sci U S A.

[74] Grifoni A., Angelo M., Sidney J., Paul S., Peters B., de Silva A.D. (2017). Patterns of cellular immunity associated with experimental infection with rDEN2Delta30 (Tonga/74) support its suitability as a human Dengue virus challenge strain. J Virol.

