# Corrigendum to ‘‘Longitudinal analysis of acute and convalescent B cell responses in a human primary dengue serotype 2 infection model’’ [EBioMedicine 41 (2019) 465–478]

**DOI:** 10.1016/j.ebiom.2020.102708

**Published:** 2020-04-03

**Authors:** Usha K. Nivarthi, Huy A. Tu, Matthew J. Delacruz, Jesica Swanstrom, Bhumi Patel, Anna P. Durbin, Stephen S. Whitehead, Kristen K. Pierce, Beth D. Kirkpatrick, Ralph S. Baric, Ngan Nguyen, Daniel E. Emerling, Aravinda M. de Silva, Sean A. Diehl

**Affiliations:** a Department of Microbiology and Immunology, University of North Carolina School of Medicine, Chapel Hill, NC 27599, USA; b Department of Microbiology and Molecular Genetics, Vaccine Testing Center, Larner College of Medicine, University of Vermont, Burlington, VT 05405, USA; c Cellular, Molecular, and Biomedical Sciences Graduate Program, University of Vermont, Burlington, VT 05405, USA; d Department of International Health, Center for Immunization Research, Johns Hopkins Bloomberg School of Public Health, Baltimore, MD 21205, USA; e Laboratory of Infectious Diseases, NIAID, National Institutes of Health, Bethesda, MD 20892, USA; f Atreca, Inc. Redwood City, California 94063, USA

The authors wish to add the following acknowledgment to their Research paper:

“The automated DNA sequencing for antibody generation was performed in Vermont Integrative Genomics Resource DNA Facility and was supported by University of Vermont Cancer Center, Lake Champlain Cancer Research Organization, and the UVM Larner College of Medicine."

