# Supplementary material for: Longitudinal analysis of acute and convalescent B cell responses in a human primary dengue serotype 2 infection model
Source: eBioMedicine. 2019 Mar 8;41:465–78. doi: 10.1016/j.ebiom.2019.02.060 (PMC6444124; doi:10.1016/j.ebiom.2019.02.060)
Supplement: Supplementary file 1 — Supplementary material [file mmc1.pdf]

Supplementary Materials

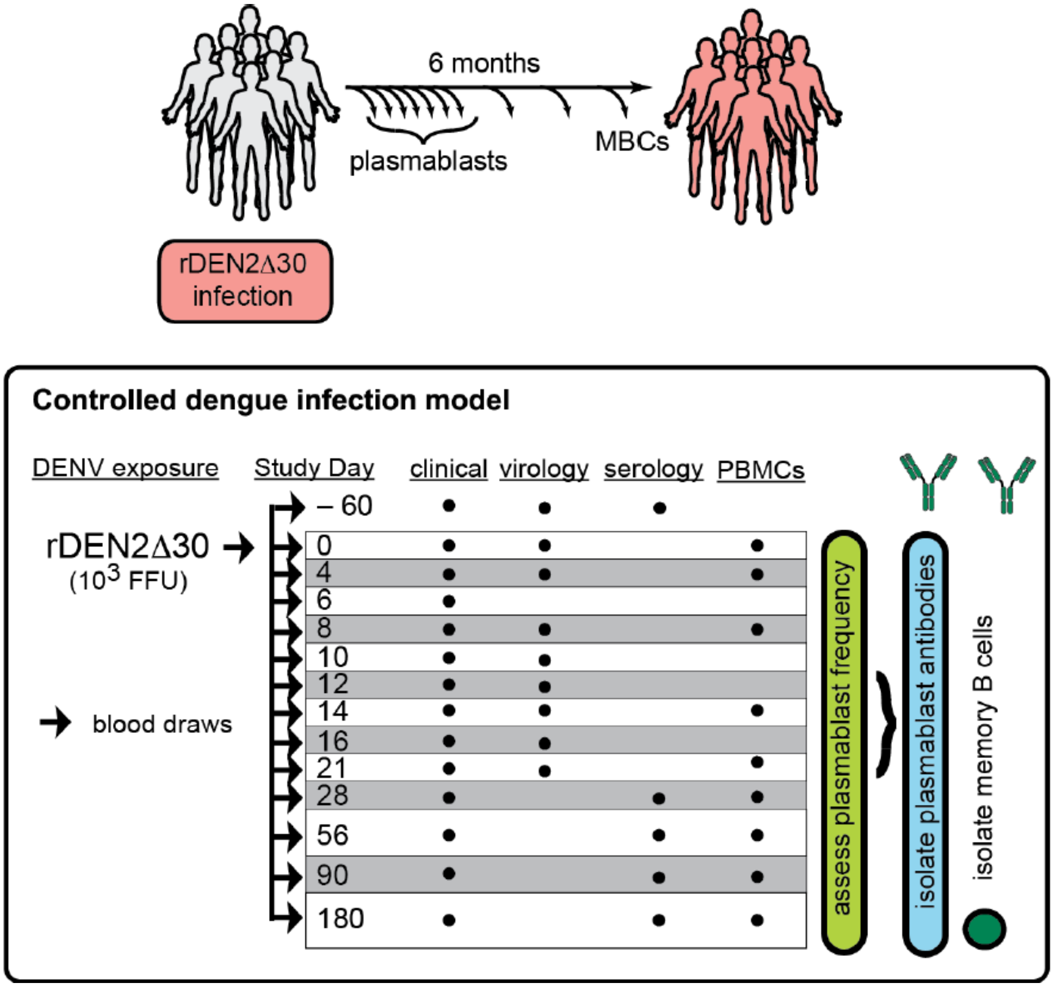

Figure S1 | Study design of controlled dengue 2 human infection. Flavivirus naïve subjects were infected with rDEN2Δ30 as the placebo arm of a vaccine/challenge trial [41]. All subjects were viremic with mean peak titer of 2.3 log_{10} FFU/mL and all subjects seroconverted to DENV2.

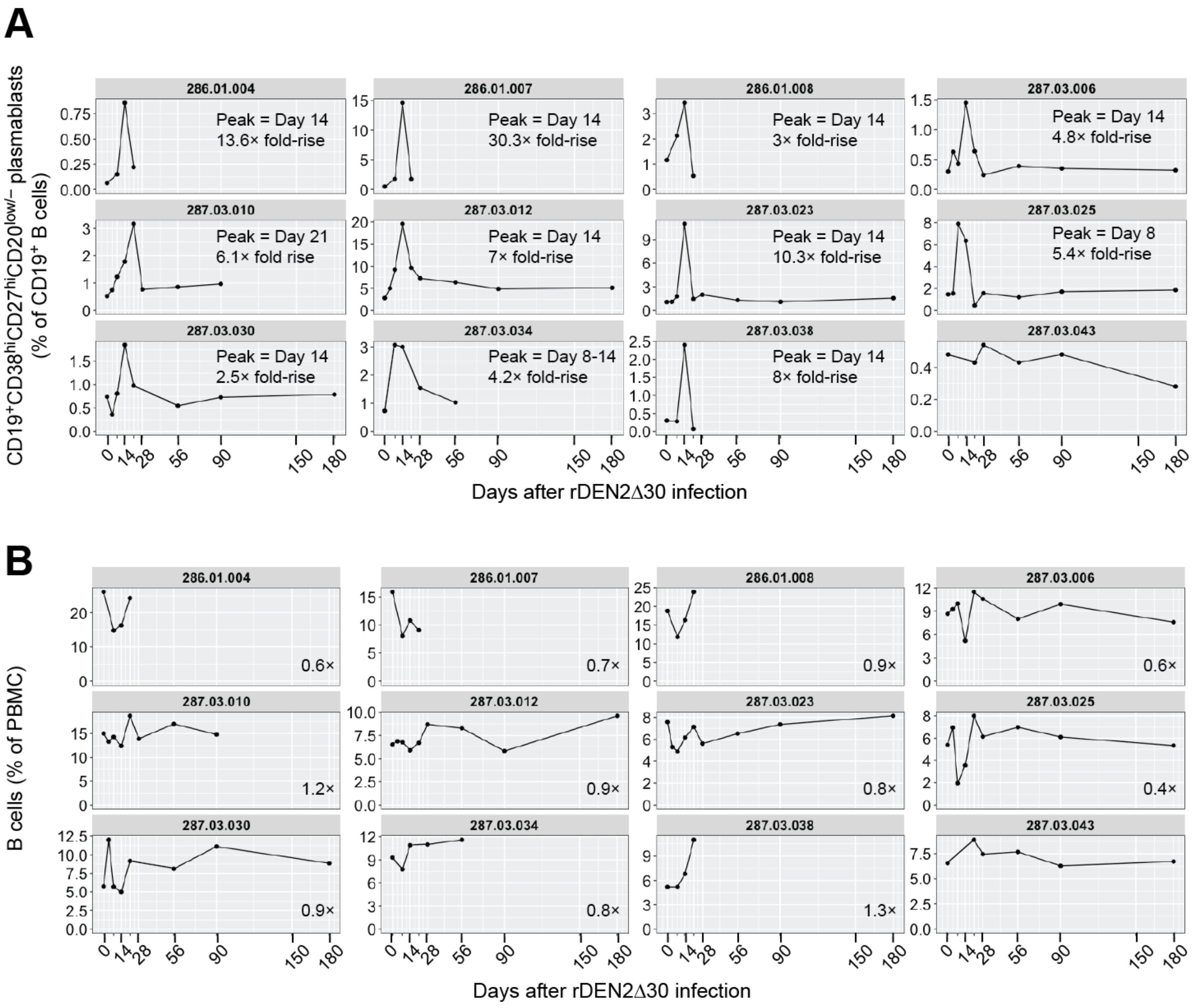

Figure S2 | Subject-specific plasmablast frequencies (as percent of total B cells) and overall B cell frequencies (as percent of live PBMC) to primary rDEN2Δ30 infection. (A) Day of peak plasmablast response and fold-rise compared to baseline (Day 0) is noted in inset (average 8.7–fold, range = 2.5–30-fold rise in plasmablast frequencies at peak). Fold-rise indicates the fold change in plasmablast frequencies measured at Day 0 to frequencies measured on day of peak response. (% plasmablasts at day of peak response divided by the % plasmablasts at Day 0). (**B**) B cell frequencies were not affected by rDEN2Δ30 infection (average 1.3-fold).

A

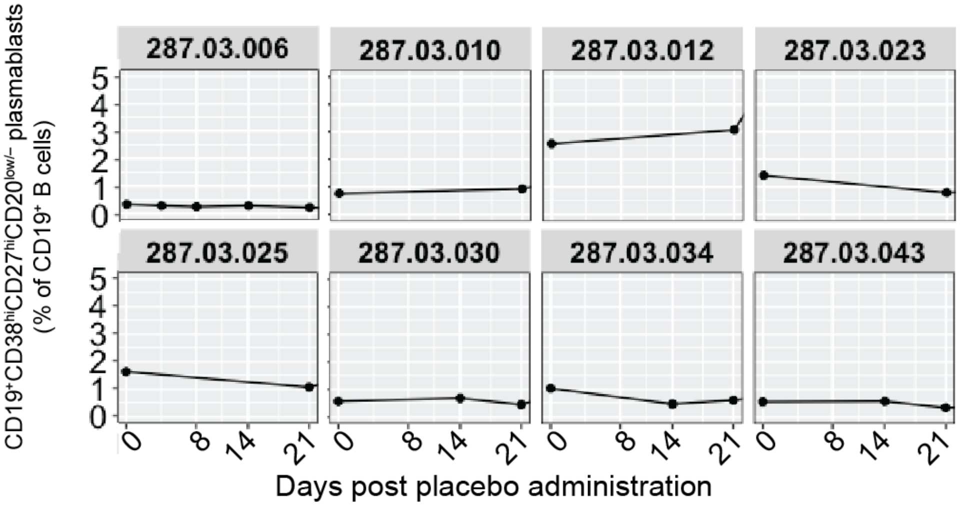

B

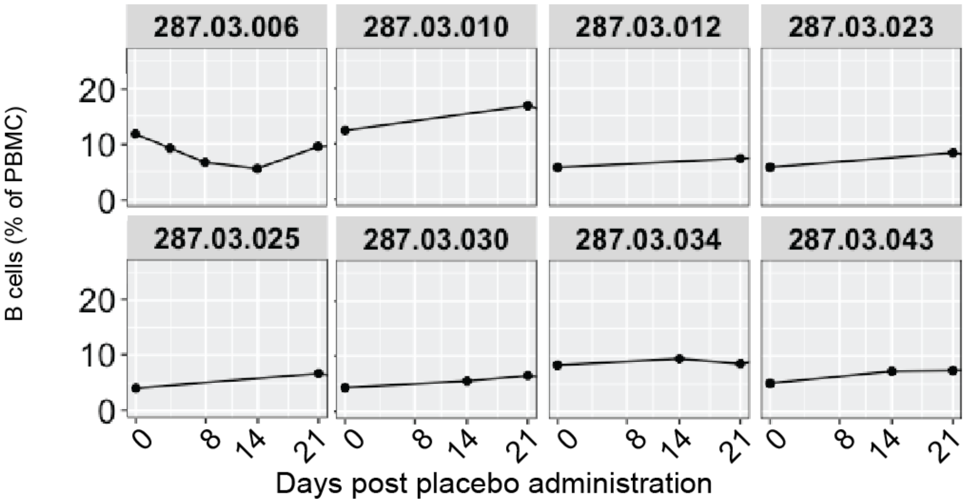

Figure S3 | Pre-challenge baseline plasmablast (A) and total B cell (B) frequencies in eight subjects prior to rDEN2Δ30 challenge. Subjects received a placebo (virus diluent) and plasmablast and B cell frequencies were monitored for a three-week period. These subjects were then later challenged with rDEN2Δ30 (in Figure S2).

Table S1 | Lineage traits of plasmablasts sequenced from three subjects infected with rDEN2Δ30.

Subject	*010*	*025*	*038*	*Total*
Plasmablast sequenced (pairs)	715	390	585	1690
Lineages detected	379	188	356	923
Lineages detected with >1 plasmablast ("expanded")	78	35	98	211
Plasmablasts in expanded lineages	414	237	327	978
Percent of all plasmablasts that are in expanded lineages	58%	61%	56%	58%

Table S2 | Lineage mapping of DENV2-binding mAbs in individual rDEN2Δ30-induced plasmablast repertoires

mAb	*010*	*025*	*038*	*Total or average[1]*
No. of PBs in repertoire	715	390	585	1690
No. of PBs in expanded lineages	414	237	327	978
No. of PBs in tested lineages	335	202	204	741
Fraction of PBs in expanded lineages that were in tested lineages	81%	85%	62%	76%
No. of PBs in DENV2+ lineages	106	141	123	370
Fraction of PBs in tested lineages that are DENV2+	32%	70%	60%	54%[1]

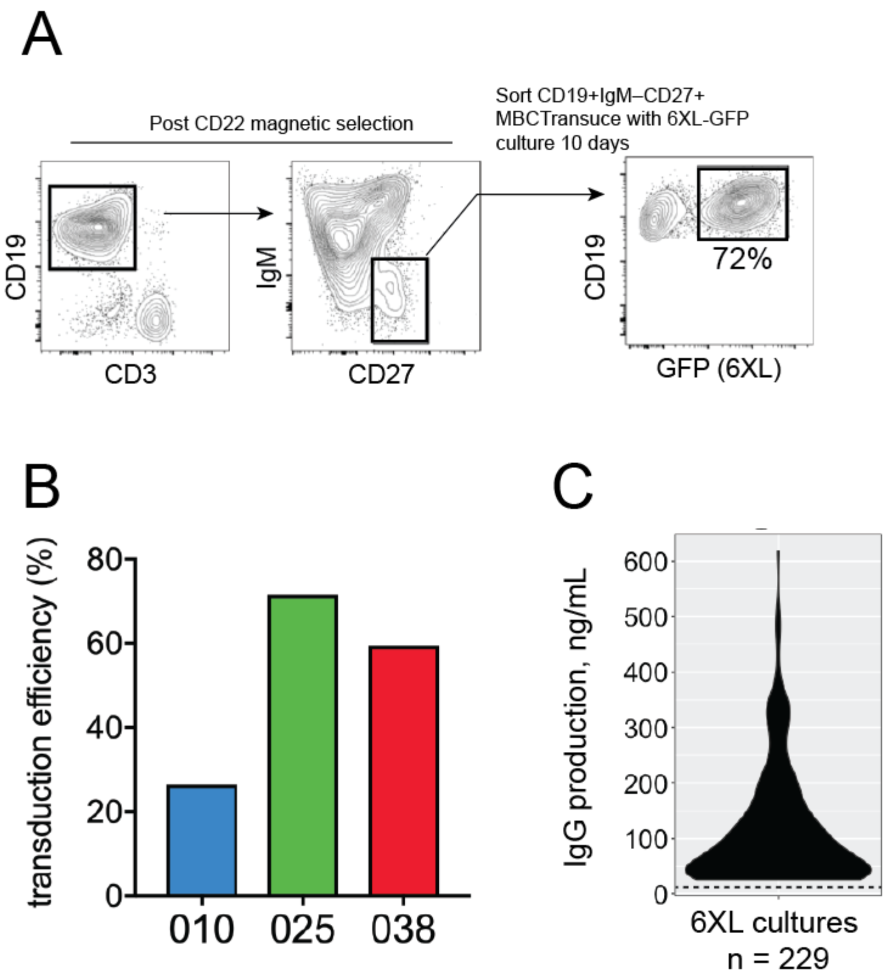

Figure S4 | Isolation and immortalization of switched peripheral blood memory B cells (**A**) representative fluorescence -assisted cell sorting of $CD19^{+}CD3^{-}IgM^{-}$ $CD27^{+}$ memory B cells that are then transduced with BCL6+Bcl-xL (6XL) retroviral vector containing a green fluorescence protein (GFP) marker which is used to assess transduction efficiencies by flow cytometry of $CD19^{+}$ B cells 10 days after transduction. (**B**) transduction efficiencies of IgM^{-} $CD27^{+}$ MBC from subjects 287.03.010, 287.03.025, and 287.03.038. (**C**) IgG production by stably-transduced 6XL+ MBC. Dashed line, limit of detection, 5 ng/mL

Table S3. Sequence and functional characteristics of *IGH/IGL* pairs that were convergent in two or more subjects infected with rDEN2Δ30.

Priority #[a]	mAb ID	Subject IDs	IGH-V			IGL-V			DENV2-binding[d]	DENV2 neutralizing[d]
			VH gene usage	CDRH3 length (AA)	% Identity[c]	VL gene usage	CDRL3 length (AA)	% Identity[c]		
1	1579	**010**<br>**038**	IGHV3-30/33[b]	17	80	IGKV1D-39	10	86	+	–
2	1652	**025**<br>**038**	IGHV3-74	12	80	IGLV3-9	10	86	+	+
4	1618	**010**<br>**025**	IGHV3-30/33	15	77	IGLV1-51	12	81	–	ND[e]
6	1581	**025**<br>**038**	IGHV5-51	16	83	IGKV4-1	10	91	–	ND
7	1619	**010**<br>**038**	IGHV1-18	17	84	IGLV2-11	11	87	–	ND
8	1582	**025**<br>**038**	IGHV3-30/33	14	80	IGKV1D-39	10	84	–	ND
10	1636	**025**<br>**038**	IGHV3-49	13	84	IGLV4-69	10	91	+	–
29	1639	**025**<br>**038**	IGHV3-30.33	18	83	IGLV1-51	12	89	NA[f]	NA[g]
42	1641	**010**<br>**025**<br>**038**	IGHV3-21	20	94	IGKV3-21	12	99	+	–
93	1674	**010**<br>**038**	IGHV1-18	12	94	IGLV2-23	11	97	+	–
95	1616	**010**<br>**038**	IGHV3-30/33	15	75	IGLV3-11	10	89	+	–
96	1617	**010**<br>**038**	IGHV3-30/33	15	98	IGLV4-1	10	97	–	ND
	**Summary** (average or proportion)			15.3	84.3%		10.7	89.8%	**6/11 (55%)**	**1/11 (9%)**

a, From Figure 2E.
b, IGHV30 and IGHV33 germline genes have a high degree of sequence identity and are not called separately
c, BLASTP (https://blast.ncbi.nlm.nih.gov/Blast.cgi?PAGE=Proteins) for entire IGHV or IGLV mature peptides between subjects
d, Binding to DENV2 by ELISA (see methods)
e, Neutralization of DENV2 by $FRNT_{50}$ assay (see methods)
f, ND, not done
g, NA, not available

Table S4 | Frequencies of total B cells and IgM^{-}CD27^{+} memory B cells in subjects infected six months prior with rDEN2Δ30 and the transduction efficiencies of these IgM^{-} CD27^{+} memory B cells with 6XL.

Subject	*% B cells in PBMC*	*% IgM–CD27^{+} MBC of B cells*	*% GFP+ after 6XL transduction*
010	12.9%	23.5%	38.9%
025	7.1%	17.8%	89.6%
038	10.1%	10.6%	78.7%

Table S5 | Summary of Variable (V) gene usage, CDRH3 sequence, and somatic hypermutation of four monoclonal DENV2-binding mAbs derived from memory B cells at 6 months after rDEN2Δ30 infection.

Poly-clonal culture ID	*Mono-clonal culture ID*	*Heavy/ Light chain*	*IGH/IGL gene usage*	*CDRH3 length (# of aa)*	*CDRH3 sequence (aa)*	*Mutation rate compared to germline*		*NEUT$_{50}$ DENV2 (ng/mL)*
						SHMs (total H+L)	non-silent (H+L)	
F6	11 clones	IgG1<br>Ig-κ	IGHV4-59/23<br>IGKV3-20.3D/10	23	ARGAARSPRRRGTLYSYPYMDVW	40 (25+15)	35 (20+15)	n.d.
B10	B10	IgG1<br>Ig-λ	IGHV3-30.33/12<br>IGLV2-14/13	12	ARGLGLVPGAYW	34 (19+15)	29 (16+13)	Negative
F11	B7	IgG4<br>Ig-κ	IGHV3-23/20<br>IGKV3-20.3D/9	20	VRGPSGGFWSGYYIGAFDSW	44 (27+17)	41 (25+16)	Negative
F5	E6	IgG1<br>Ig-λ	IGHV1-69/17<br>IGLV2-11/11	17	AISGAAAASPYYYLDVW	28 (23+5)	26 (21+5)	35-100

n.d. = not done.

Table S6 | Functional properties of plasmablast-, MBC-, and serum-derived antibodies in three subjects infected with rDEN2Δ30.

		Plasmablasts	***MBC***	***Plasma (Neutralizing antibodies)***		
				Tonga	*NGC*	*S16803*
Frequencies of DENV specific B cell clones	*287.03.010*	38%	0.32%			
	287.03.025	44%	0.73%			
	287.03.038	51%	0.27%			
Strain specific neutralization titers for serum	*287.03.010*			45	410	152
	287.03.025			1254	3734	3925
	287.03.038			361	194	304
TS	*287.03.010*	57%	54%			
	287.03.025	43%	26%			
	287.03.038	85%	100%			
CR	*287.03.010*	43%	46%			
	287.03.025	57%	74%			
	287.03.038	15%	0%			
% TS Neutralizing antibodies in serum	*287.03.010*					100%
	287.03.025					68%
	287.03.038					98%
% CR Neutralizing antibodies in serum	*287.03.010*					0%
	287.03.025					32%
	287.03.038					2%
